# Phase angle and rectus femoris cross-sectional area as predictors of severe malnutrition and their relationship with complications in outpatients with post-critical SARS-CoV2 disease

**DOI:** 10.3389/fnut.2023.1218266

**Published:** 2023-11-21

**Authors:** Víctor J. Simón-Frapolli, Isabel M. Vegas-Aguilar, Rocío Fernández-Jiménez, Isabel M. Cornejo-Pareja, Ana M. Sánchez-García, Pilar Martínez-López, Pilar Nuevo-Ortega, Carmen Reina-Artacho, María A. Estecha-Foncea, Adela M. Gómez-González, María B. González-Jiménez, Elma Avanesi-Molina, Francisco J. Tinahones-Madueño, José M. García-Almeida

**Affiliations:** ^1^Department of Endocrinology and Nutrition, Virgen de la Victoria University Hospital, Málaga, Spain; ^2^Instituto de Investigación Biomédica de Málaga, Virgen de la Victoria University Hospital, Málaga, Spain; ^3^Facultad de Medicina, University of Málaga, Málaga, Spain; ^4^Department of Critical Care, Virgen de la Victoria University Hospital, Málaga, Spain; ^5^Department of Physical Medicine and Rehabilitation, Virgen de la Victoria University Hospital, Málaga, Spain; ^6^Department of Mental Health, Virgen de la Victoria University Hospital, Málaga, Spain; ^7^Department of Endocrinology and Nutrition, Hospital Quirónsalud, Málaga, Spain

**Keywords:** malnutrition, morphofunctional assessment, phase angle, ultrasound of rectus femoris muscle, reduced muscle mass, post-critical SARS-CoV2 disease, muscle mass

## Abstract

**Background and aims:**

The diagnosis of malnutrition in post-critical COVID-19 patients is challenging as a result of the high prevalence of obesity, as well as the variability and previously reported inconsistencies across currently available assessment methods. Bioelectrical impedance vector analysis (BIVA) with phase angle (PhA) and nutritional ultrasound (NU^®^) are emerging techniques that have been proven successful in assessing body composition with high precision in previous studies. Our study aims to determine the performance and usefulness of PhA and rectus femoris cross-sectional area (RF-CSA) measurements in assessing body composition as part of the full routine morphofunctional assessment used in the clinical setting, as well as their capacity to predict severe malnutrition and to assess complications and aggressive therapy requirements during recent intensive care unit (ICU) admission, in a cohort of post-critically ill COVID-19 outpatients.

**Methods:**

This prospective observational study included 75 post-critical outpatients who recovered from severe COVID-19 pneumonia after requiring ICU admission. Correlations between all the morphofunctional parameters, complications, and aggressive therapy requirements during admission were analyzed. Multivariate logistic regression analysis and ROC curves were provided to determine the performance of NU^®^ and PhA to predict severe malnutrition. Differences in complications and aggressive therapy requirements using the cutoff points obtained were analyzed.

**Results:**

In total, 54.7% of patients were classified by Subjective Global Assessment (SGA) as SGA-B and 45.3% as SGA-C, while 78.7% met the Global Leadership Initiative of Malnutrition (GLIM) criteria. PhA correlates positively with body cell mass/height (BCM/h) (*r* = 0.74), skeletal muscle index (SMI) (*r* = 0.29), RF-CSA (*r* = 0.22), RF-Y axis (*r* = 0.42), and handgrip strength (HGS) assessed using dynamometry (*r* = 0.42) and the Barthel scale (*r* = 0.29) and negatively with ICU stay (*r* = −0.48), total hospital stay (*r* = −0.57), need for invasive mechanical ventilation (IMV) (*r* = −0.39), days of IMV (*r* = −0.41), need for tracheostomy (*r* = −0.51), and number of prone maneuvers (*r* = −0.20). RF-CSA correlates positively with BCM/h (*r* = 0.41), SMI (*r* = 0.58), RF-Y axis (*r* = 0.69), and HGS assessed using dynamometry (*r* = 0.50) and the Barthel scale (*r* = 0.15) and negatively with total hospital stay (*r* = −0.22) and need for IMV (*r* = −0.28). Cutoff points of PhA < 5.4° and standardized phase angle (SPhA) < −0.79 showed good capacity to predict severe malnutrition according to SGA and revealed differences in ICU stay, total hospital stay, number of prone maneuvers, need for IMV, and need for rehabilitation, with statistical significance (*p* < 0.05). An RF-CSA/h < 2.52 cm^2^/m (for men) and <2.21 cm^2^/m (for women) also showed good performance in predicting severe malnutrition and revealed differences with statistical significance (*p* < 0.05) in ICU stay and total hospital stay.

**Conclusion:**

More than 75% of the post-critical COVID-19 survivors had malnutrition, and approximately half were obese. PhA, SPhA, RF-CSA, and RF-CSA/h, when applied to the assessment of body composition in post-critical COVID-19 patients, showed moderate-to-high correlation with other morphofunctional parameters and good performance to predict severe malnutrition and to assess complications and aggressive therapy requirements during ICU admission. Besides being readily available methods, BIVA and NU^®^ can help improve the morphofunctional assessment of malnutrition in post-critical COVID-19 survivors; however, more studies are needed to assess the performance of these methods in other populations.

## 1 Introduction

Coronavirus 2019 (COVID-19) is an infectious disease of recent appearance in our environment that serves as a model of acute inflammatory disease/infection with severe immune response (1). Many had severe hypoxemia secondary to bilateral pneumonia and required admission to intensive care (2), and this led to a significant increase in the rates of severe malnutrition, functional deterioration, respiratory distress, and other psychophysical complications derived from both the evolution of the disease itself and prolonged stay in the ICU (3). Thus far, few studies have evaluated functional aspects such as motor sequelae with impact on the quality of life (QoL) (4), perceived level of fatigue (5), exercise intolerance, and limitations in physical capacity and their influence on psychological health (6), as well as sequelae in nutritional status (7), making a multidisciplinary approach to these patients necessary due to their complexity (8).

It is well-known that critically ill patients with severe acute COVID-19 pneumonia are at high risk of disease-related malnutrition and sarcopenia (9–14). Malnutrition is often underdiagnosed and undertreated but is a major health risk for patients (15, 16). However, the effects of COVID-19 on strength and muscle mass loss in these patients have not been extensively studied in this population, and there is limited evidence in this field (17). However, new data from the ESPEN-WHO Europe have revealed that impaired body composition, specifically low measured muscle mass, is a predictor of poor clinical outcomes in COVID-19 patients (18–20). Consistent with these findings, a recent meta-analysis confirmed an association between low skeletal muscle mass and COVID-19 severity and mortality (21).

For the evaluation of nutritional status, in recent years, a series of emerging parameters such as BIVA with PhA, NU^®^, and HGS by dynamometry have been added to the classical analytical and anthropometric parameters, which allow a more accurate morphofunctional analysis as they not only refine more in terms of body composition but also incorporate the analysis of muscle function and cellular health (22). BIVA is a technique that characterizes changes in body composition based on the human body's ability to transmit an electrical current, providing bioelectrical parameters such as PhA, which is a global marker reflecting a patient's nutritional and inflammatory status, useful as an independent prognostic factor in a wide variety of pathologies, including acute COVID-19 pneumonia (18).

NU^®^ is a new technique that uses ultrasound to discriminate, evaluate, and measure the rectus femoris (RF) lean and adipose tissue, due to the good correlation between this muscle and strength functional performance tests, and the abdominal adipose tissue and thus extrapolate the body composition of the organism (23–25).

However, despite the emergence of these new assessment tools, we do not have reliable cutoff points for discriminating severe malnutrition from moderate malnutrition, especially in post-critical and obese patients. Therefore, our study aimed to determine the performance of PhA and RF-CSA in predicting severe malnutrition and their capacity to assess complications and aggressive therapy requirements during admission in a cohort of post-critically ill COVID-19 outpatients. A secondary objective was to analyze the correlation of Virge and RF-CSA with the rest of the morphofunctional assessment parameters to reinforce their clinical applicability.

## 2 Materials and methods

### 2.1 Study design

This prospective observational study included 75 patients who had been admitted to the intensive care unit of Virgen de la Victoria University Hospital from April 2020 to October 2021 for severe COVID-19 pneumonia. All patients were diagnosed with COVID-19 pneumonia during admission, according to the World Health Organization (WHO) Interim Guideline, and with SARS symptoms by nasopharyngeal specimen upon admission using real-time reverse transcriptase-polymerase chain reaction assay. Patients admitted to the ICU were those eligible for aggressive measures, with oxygen requirements >15 liters per minute and having PaO2/FiO2 < 200.

All subjects received the informed consent form before participating in the study at the time of hospital discharge. The study was conducted in accordance with the Declaration of Helsinki, and the protocol was approved by the Ethics Committee of Virgen de la Victoria University Hospital (PI-20-321, September 2021). All patients included in our study met the inclusion criteria (age over 18 years; recent admission to ICU for severe COVID-19 pneumonia, defined as respiratory distress with ≥30 breaths/min or resting O2 saturation ≤ 93%; consent to participate in the study by means of accepted informed consent; and possibility of a consultation assessment within 14–21 days after hospital discharge) and had no exclusion criteria (pathology or baseline condition not requiring admission to ICU; refusal to participate; patients from other hospitals whose follow-up was complicated; patients residing abroad; impossibility of measurement by NU^®^ or BIVA; amputations, extensive skin lesions or local hematomas, diseases that severely interfere with morphofunctional assessment such as muscular dystrophies, end-stage renal failure, decompensated heart failure, or severe ascites).

Patients were evaluated in a specific medical nutrition consultation 14–21 days after hospital discharge. Socio-demographic and clinical characteristics of the patients were collected, as well as a complete morphofunctional assessment using the analyses detailed in the following sections. Subsequently, they were classified into nutritional risk categories according to both SGA and GLIM criteria, and differences in morphofunctional variables were analyzed.

### 2.2 Diagnostic tools for malnutrition

Between 14 and 21 days after hospital discharge, patients were classified using the following diagnostic tools:

Subjective Global Assessment (SGA) is a method developed by Detsky et al. (26) based on data from clinical history (weight loss during the last 6 months, dietary intake at the time of assessment compared to usual, presence of gastrointestinal symptoms, functional capacity, and metabolic stress) and physical examination (loss of subcutaneous fat, decreased muscle mass, malleolar or sacral edema, and ascites). For many authors, it is the gold standard for validating new methods of screening and/or assessing disease-related malnutrition (27–30).

The criteria established by the Global Leadership Initiative on Malnutrition (GLIM) allowed for a more exhaustive nutritional assessment by including the evaluation of muscle mass loss and disease burden/inflammation. This GLIM initiative for the diagnosis of malnutrition is based on the presence of a phenotypic criterion (including percentage weight loss, body mass index, and muscle mass loss determined by validated body composition measurement techniques, such as appendicular skeletal muscle mass index (ASMMI) < 7 kg/m^2^ in men or < 5.5 kg/m^2^ in women and fat-free mass index (FFMI) < 17 kg/m^2^ in men or < 15 kg/m^2^ in women) and an etiologic criterion such as reduced food intake or assimilation or the presence of inflammation, usually associated with a C-reactive protein (CRP) > 10 mg/L. According to these criteria, moderate malnutrition according to GLIM is defined by weight loss of 5–10% in the last 6 months or 10–20% after 6 months and body mass index (BMI) < 20 kg/m^2^ in < 70 years or < 22 kg/m^2^ in ≥70 years or mild-to-moderate muscle mass loss according to validated techniques. Severe malnutrition is defined as weight loss of >10% in 6 months or >20% in more than 6 months and BMI < 18.5 kg/m^2^ if < 70 years or < 20 kg/m^2^ if ≥70 years or severe muscle mass loss according to validated and poorly available techniques as the lean mass index using dual-energy absorptiometry (22, 31).

### 2.3 Clinical variables

The following clinical variables were collected: age, sex, habitual weight, weight at discharge, percentage of weight loss, BMI, and cardiovascular risk factors (e.g., medical history of diabetes, hypertension, and dyslipidemia). The following complications were compiled: ICU stay, total hospital stay, hypoxemic respiratory failure requiring home oxygen therapy, referral to rehabilitation upon discharge, low Barthel scale score, and low FACIT-F scale score. Furthermore, the need for various therapies during admission such as corticosteroid treatment, IMV, tracheostomy, and number of prone maneuvers was gathered.

The FACIT Fatigue Scale is a validated tool for assessing fatigue in patients. Comprising just 13 items, the FACIT Fatigue Scale is a concise and easy tool that assesses an individual's fatigue levels during their daily activities over the past 7 days. Respondents rate their level of fatigue on a 5-point Likert scale, ranging from 0 (very much fatigued) to 4 (not at all fatigued). The total scores on this scale can vary from 0 to 52, with higher scores indicating lower levels of fatigue (32).

### 2.4 Body composition analysis

#### 2.4.1 Phase angle by BIVA

Whole-body bioelectrical impedance measurements were obtained in all patients using a 50 kHz phase-sensitive impedance analyzer [BIA 101 whole-body bioimpedance vector analyzer (AKERN, Pontassieve, Italy)] with tetrapolar 800 mA wearable electrodes placed on the right hand and foot. All patients waited 5 min in supine position before BIVA measurements were taken. The body consists of complex circuits composed of resistance (Rz) and reactance (Xc) elements which, when stimulated with an alternating current, experience a frequency-dependent current delay with respect to the voltage flow. These raw impedances Rz and Xc give PhA [PhA = arc tangent (Xc/*R*) × 180°/π]. By definition, PhA is positively associated with tissue reactance (related to cell mass function, integrity, and composition) and negatively associated with resistance, which is mostly dependent on the degree of tissue hydration (33).

Individual SPhA value was determined from the sex- and age-matched reference population value by extracting the reference PhA value from the patient's observed PhA and then dividing the result by the respective reference standard deviation (SD) by age and sex.

Bioelectrical parameters were analyzed to estimate body composition, such as fat mass (FM), fat mass percentage (FM%), fat-free mass (FFM), FFMI, body cell mass (BCM), BCM/h, appendicular skeletal muscle mass (ASMM), ASMMI, SMI, total body water (TBW), extracellular body water (ECW), Na/K exchange, and hydration percentage (TBW/FFM).

Interpretation of impedance value is carried out using BIVA, which assesses patients through the direct determination of the impedance vector, without depending on body weight, equations, or models, as is the case with conventional BIA. This method was introduced by Piccoli et al. (34), where Rz and Xc, standardized for height, are plotted as point vectors on the so-called resistance–reactance graph (RzXc graph), which consists of a plane showing the tolerance ellipses (50, 75, and 95% percentiles), defined according to the vector distribution of the healthy reference population.

#### 2.4.2 Nutritional ultrasound^®^ evaluation of the rectus femoris (RF) and abdominal adipose tissue

**Figure 1:**
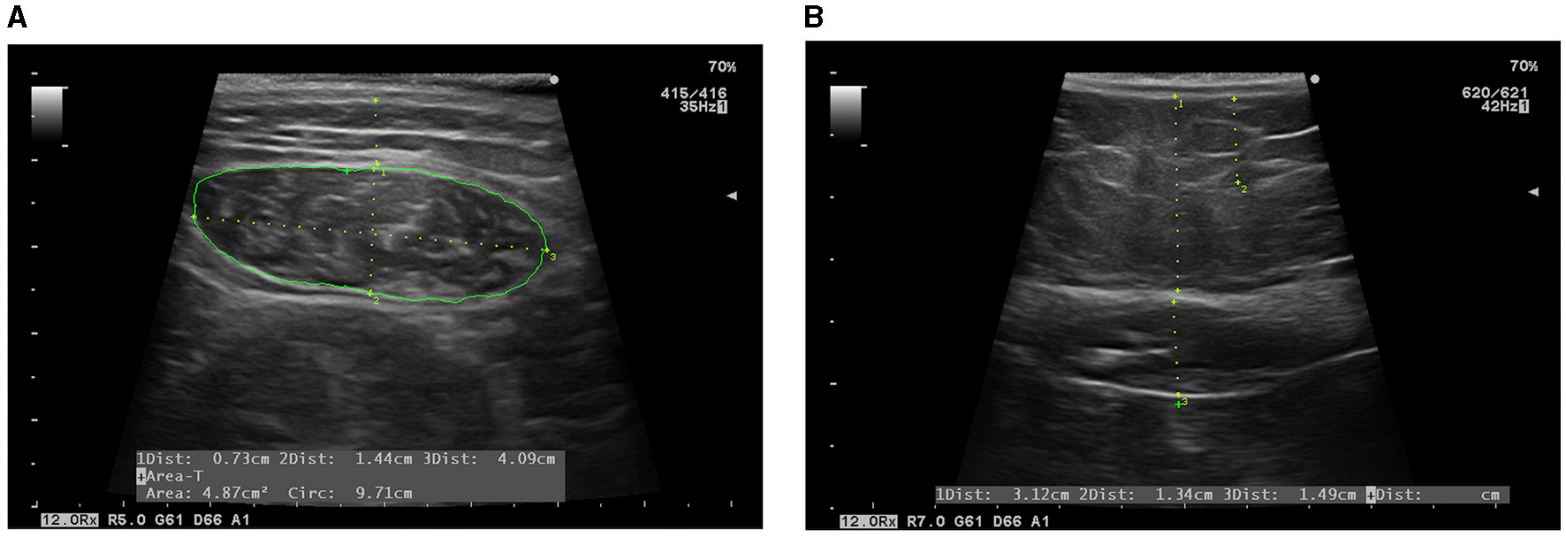
Evaluation by NU^®^. **(A)** Evaluation of RF: L-SAT (1), RF-Y axis (2), RF-X axis (3), RF-CIR, and RF-CSA (green line) are measured. **(B)** Evaluation of abdominal adipose tissue: T-SAT (1), superficial subcutaneous abdominal adipose tissue, and VAT (3) are measured.

A comprehensive nutritional ultrasound assessment was performed using a HITACHI ALOKA F37 ultrasound scanner with an Aloka UST-5413 Linear Array transducer with a frequency range of 5.0–10 MHz in B-mode in transverse position (Hitachi Europe, Stoke Poges Ltd., Buckinghamshire, UK). During the assessment, patients should lie in supine position with arms supinated and knees extended and relaxed to full extension. This should be carried out by qualified professionals in the field, using an appropriate water-soluble transmission gel to ensure acoustic contact without indenting the skin surface, and aligned perpendicular to the longitudinal and transverse axes of the RF to obtain the cross-sectional image. Three images of the right RF muscle were recorded, and the average measurements were estimated for accuracy. The acquisition site was located two-thirds of the length of the femur, measured between the superior pole of the patella and the anterosuperior iliac spine. RF-CSA in cm^2^, muscle circumference (RF-CIR) in cm, muscle thickness (or RF-*Y* axis) in cm, RF-*X* axis in cm, and leg subcutaneous adipose tissue (L-SAT) in cm were measured in the transverse axis (35), as represented in Figure 1. Cross-sectional area/height (RF-CSA/h) and cross-sectional area/weight (RF-CSA/w) were also obtained. An increasing number of studies have been published on this technique (6, 36).

**Figure 2:**
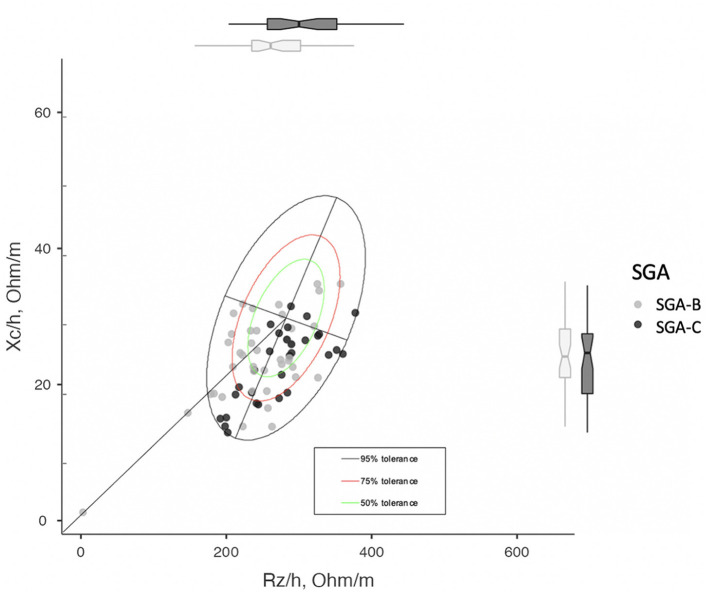
RzXc graph divided by SGA. It can be seen how SGA-C patients tend to fall in the lower right quadrant with respect to the SGA-B, which indicates a higher degree of hydration and a lower cell mass.

In addition, subcutaneous and preperitoneal visceral adipose tissues were also assessed at the abdominal level. The acquisition site was measured between the xiphoid and the umbilicus, with the patient in supine position. This should also be performed by experienced professionals in the field. Images were taken during unforced expiration, in a transverse axis, and aligned perpendicular to the skin. Total subcutaneous abdominal adipose tissue (T-SAT) was obtained. Total visceral adipose tissue (VAT) was determined by measuring the distance between the boundary of the parietal peritoneum and the inner aspect at the junction of the two rectus abdominal muscles, as shown in Figure 2. To minimize measurement variability, three measurements were carried out, and the mean value was recorded (37).

### 2.5 Functional and muscle strength assessment

HGS was measured using the JAMAR Dynamometer (J A Preston Corporation, New York, NY, USA), and both hands were assessed. Three measurements were taken, and the mean was calculated and compared with published population reference data used as cutoff points (38). The timed get-up-and-go (TUG) test was used as an assessment of functional status. To measure participants' TUG time, a colored tape was marked 3 m from an armless chair in which the participants were seated. Participants were asked to walk 3 m, turn around the marked tape, and return to the chair as fast as possible. A stopwatch was started when the participant got up from the chair and stopped when they sat down. At least one practice run was performed before the test. TUG value over 12 s is considered a practical cutoff for a low performance (39), whereas values > 20 s indicate severe sarcopenia (40). The 6-min walk test (6 MWT) is a submaximal cardiorespiratory stress test that consists of measuring the maximum distance that a person can walk on a flat surface in 6 min (41, 42).

### 2.6 Analytical variables

Specific biomolecular markers, such as vitamin D, prealbumin, and the CRP/prealbumin ratio, were measured to assess nutrition and inflammation. Prealbumin is much more sensitive than albumin to changes in whole-body protein status and is not affected by hydration status. In addition, prealbumin is a negative acute phase reactant protein, whose level decreases in malignancy and inflammation and should therefore be corrected by CPR. Its association with CRP levels, a marker of inflammation in the body, may increase its interest as a predictor of morbimortality, and of nutritional/inflammatory changes. CRP/prealbumin is independently associated with hospital mortality (19).

### 2.7 Statistical analysis

Statistical analyses of the data were performed using Jamovi program (version 1.6.23.0 for Mac, Jamovi, Spain).

Descriptive statistics were used to characterize our cohort of patients, both globally and according to nutritional risk categories. Normality of the variables was assessed using the Kolmogorov–Smirnov test. Baseline characteristics were expressed as mean (interquartile range) ± SD for continuous variables and as absolute value (proportion) for categorical variables. Continuous variables were compared with Student's *t*-test and ANOVA (with the addition of Tukey's multiple comparison test to study which specific groups showed differences), or with Mann–Whitney and Kruskal–Wallis *U*-test, depending on their distribution. Categorical variables were compared using the chi-squared test (and Fisher's exact test when required). These analyses were carried out with a confidence interval of 95%. The relationship was also analyzed using Pearson's or Spearman's correlation models depending on their distribution, and a correlation heatmap as a graphical tool that displays the correlation between multiple variables as a color-coded matrix was generated. A *p*-value of < 0.05 was considered statistically significant.

The diagnostic performance of PhA, SPhA, BCM, BCM/h, FFMI, ASMM, ASMMI, RF-CSA, RF-CSA/h, RF-CSA/w, and RF-Y axis to assess severe malnutrition was evaluated with the area under the curve (AUC) by constructing a plot of sensitivity vs. specificity. ROC curves and Youden's index were used to determine the optimal cutoff points. Using a binomial logistic regression model, a multivariate analysis was performed to evaluate the predictors of severe malnutrition characterized by SGA-C. The variables in which a statistical association was observed in the univariate analysis were entered into the multivariate analysis.

Finally, we analyzed whether the cutoff points obtained by ROC curves showed statistically significant differences in terms of complications and aggressive therapy requirements during admission, using Student's *t*-test between the two subgroups above and below the cutoff point.

## 3 Results

### 3.1 Baseline characteristics of post-critical COVID-19 outpatients

A total of 360 patients were admitted to the intensive care unit of the Hospital Virgen de las Victoria from April 2020 to October 2021. A total of 75 patients were referred to the Nutrition Unit as part of the post-COVID multidisciplinary consultation. Of the remaining 285 patients, 127 patients died during hospital admission, 74 patients did not meet the inclusion criteria (28 were transferred from other hospitals, 45 patients were foreign patients, and only 1 patient did not wish to participate in the follow-up), and 84 patients were lost to follow-up after hospital discharge.

The clinical and demographic characteristics of the participants are included in Table 1. The mean age was 62 ± 12 y, with a mean BMI of 31.1 ± 6.4 kg/m^2^. In total, 61.8% were obese (using BMI ≥ 30 kg/m^2^). The mean weight at discharge was 99.1 ± 24.7 kg, with a loss of 10.7 ± 1.8% with respect to usual weight. A total of 58 patients (73.3%) were men. Regarding other comorbidities, 28.0% of the patients were previously diabetic, 50.7% hypertensive, and 34.7% dyslipidemic. A total of 57.3% of patients required IMV during admission, with a mean duration of 27.7 ± 21.4 days. The respiratory therapy used prior to orotracheal intubation was high-flow nasal oxygen therapy (ONAF) in all cases. Tracheostomy was performed in 62.8% of patients. The mean number of prone maneuvers performed in each patient during admission was 2.41 ± 2.5, and more than 95% required high doses of corticosteroid therapy. The mean ICU stay was 22.7 ± 20.1 days, while the total hospital stay was 48.3 ± 44.9 days. A total of 52% of patients required home oxygen therapy after discharge, while 89.3% were referred to a specific rehabilitation program during admission and after discharge. At the post-discharge visit, 53.9% of patients were classified as SGA-B and 46.1% as SGA-C, while 78.9% met malnutrition criteria according to GLIM (28.9% as moderate malnutrition and 50% as severe malnutrition). Differences in baseline characteristics between these categories are shown in Tables 1, 2. GLIM phenotypic criteria distribution in malnourished patients is shown in Table 3. In addition, baseline characteristics and morphofunctional assessment are divided by sex (Supplementary Table 1).

**Table 1:** Baseline characteristics of the population of study according to nutritional status (SGA).

	**All**	**SGA-B**	**SGA-C**	***p*-value**
	***N** = **75***	***N** = **41***	***N** = **34***	
**Demographic variables**				
Age (years)	62 ± 12.0	58.4 ± 12.1	65.9 ± 9.7	0.013^*^
Diabetes mellitus (%)	21 (28.0)	11 (26.8)	10 (29.4)	0.804
Hypertension (%)	38 (50.7)	22 (43.6)	16 (47.1)	0.589
Dyslipidemia (%)	26 (34.7)	15 (36.6)	11 (32.3)	0.701
Obesity by BMI ≥ 30 kg/m^2^ (%)	34 (45.3)	23 (56.1)	11 (32.4)	0.123
Obesity by FM ≥ 30% (men) or ≥ 40% (women)	36 (48.0)	19 (46.3)	17 (50.0)	0.916
**Nutritional status**				
Usual weight (kg)	99.1 ± 24.7	105.2 ± 25.1	91.6.1 ± 21.9	0.016^*^
Discharge weight (kg)	90.5 ± 21.1	95.1 ± 23.1	78.9 ± 16.9	< 0.001^*^
Weight loss (%)	10.9 ± 8.31	9.3 ± 7.6	12.9 ± 8.7	0.058
BMI (kg/m^2^)	31.1 ± 6.4	33.2 ± 8.1	28.5 ± 4.6	0.001^*^
Malnutrition (GLIM):				0.336
Normonutrition (%)	16 (21.1)	10 (24.4)	6 (23.5)	
Stage 1/moderate (%)	22 (28.9)	12 (29.2)	10 (29.4)	
Stage 2/severe (%)	37 (49.3)	19 (43.9)	18 (52.9)	
**Complications and need for aggressive therapies**				
ICU stay (days)	22.7 ± 20.1	18.5 ± 19.4	28.1 ± 18.7	0.010^*^
Hospital stay (days)	48.3 ± 44.9	40.5 ± 43.0	57.7 ± 45.5	0.011^*^
Invasive mechanical ventilation (%)	43 (57.3)	19 (46.3)	24 (70.6 )	0.035^*^
Invasive mechanical ventilation (days)	27.7 ± 21.4	24.4 ± 2034	30.7 ± 19.6	0.154
Tracheostomy (%)	27 (62.8)	9 (47.3)	18 (75.0)	0.063
Maneuvers prone (n)	2.41 ± 2.5	2.44 ± 2.3	2.36 ± 1.8	0.734
Corticosteroid therapy (%)	74 (98.7)	40 (97.5)	34 (100.0)	0.359
Home oxygen therapy after hospital discharge (%)	39 (52.0)	19 (46.3)	14 (41.2)	0.654
Rehabilitation after discharge (%)	67 (89.3)	36 (87.8)	31 (91.2)	0.887
FACIT scale	40.1 ± 13.1	38.9 ± 14.7	41.4 ± 10.9	0.943
Barthel scale	92.5 ± 17.4	92.2 ± 16.7	92.8 ± 18.3	0.982

Data are expressed as mean ± standard deviations or percentages. Groups were divided according to SGA. Asterisk indicates a significant difference between the SGA-A and SGA-B groups, according to Student's *t*-test (or Mann–Whitney test) (^*^p < 0.05). The chi-squared test (or Fisher's exact test) was used for variables expressed as percentages (^*^p < 0.05).

BMI, body mass index; FM, fat mass; ICU, intensive care unit.

**Table 2:** Baseline characteristics of the population of study according to nutritional status (GLIM).

	**All**	**Normonutrition**	**Moderate malnutrition**	**Severe malnutrition**	***p*-value**
	***N** = **75***	***N** = **13***	***N** = **42***	***N** = **20***	
**Demographic variables**					
Age (years)	62 ± 12.0	58.7 ± 12.3	62.9 ± 11.0	60.85 ± 14.7	0.539
Diabetes mellitus (%)	21 (28.0)	3 (23.1)	14 (33.3)	4 (20.0)	0.501
Arterial hypertension (%)	38 (50.7)	9 (69.2)	18 (42.8)	11 (55.0)	0.227
Dyslipidemia (%)	26 (34.7)	5 (38.4)	13 (30.9)	8 (40.0)	0.745
Obesity by BMI ≥ 30 kg/m^2^ (%)	34 (45.3)	8 (61.5)	14 (33.3)	13 (65.0)	0.123
Obesity by FM ≥ 30% (men) or ≥ 40% (women)	36 (48.0)	7 (53.8)	20 (47.6)	9 (45.0)	0.916
**Nutritional status**					
Usual weight (kg)	90.5 ± 21.1	106.6 ± 25.0	93.6.1 ± 26.9	105.7 ± 18.5	0.100
Discharge weight (kg)	99.1 ± 24.7	101.7 ± 23.3^a^	82.2 ± 21.9^a^	90.4 ± 17.6	0.010^*a^
Weight loss (%)	10.9 ± 8.31	3.3 ± 1.6^a, b^	11.0 ± 9.4^a^	15.3 ± 4.0^b^	< 0.001^*b^
BMI (kg/m^2^)	31.1 ± 6.4	35.6 ± 8.4^a^	28.8 ± 5.1^a, c^	32.4 ± 5.3^c^	0.006^*^
Malnutrition (SGA):					0.877
SGA-A (%)	0 (0.0)	0 (0.0)	0 (0.0)	0 (0.0)	
SGA-B (%)	40 (53.3)	9 (69.2)	19 (47.6)	12 (60.0)	
SGA-C (%)	35 (46.1)	4 (30.8)	23 (54.7)	8 (40.0)	
**Complications and need for aggressive therapies**					
ICU stay (days)	22.7 ± 20.1	12.8 ± 10.8^a^	26.3 ± 22.4^a^	21.2 ± 17.7	0.018^*a^
Hospital stay (days)	48.3 ± 44.9	27.1 ± 12.7^a^	59.3 ± 54.5^a^	38.8 ± 28.2	0.003^*a^
Invasive mechanical ventilation (%)	43 (57.3)	4 (30.7)^a^	29 (69.0)^a^	10 (50.0)	0.038^*a^
Invasive mechanical ventilation (days)	27.7 ± 21.4	17.2 ± 14.4	27.9 ± 23.6	31.1 ± 18.1	0.304
Tracheostomy (%)	27 (62.8)	2 (15.4)	18 (42.8)	7 (35.0)	0.775
Maneuvers prone (*n*)	2.41 ± 2.5	1.23 ± 1.9	2.66 ± 2.3	2.65 ± 2.9	0.139
Corticosteroid therapy (%)	74 (98.7)	13 (100.0)	42 (100.0)	19 (95.0)	0.248
Home oxygen therapy after hospital discharge (%)	39 (52.0)	7 (53.8)	19 (45.2)	7 (35.0)	0.550
Rehabilitation after discharge (%)	67 (89.3)	11 (84.6)	38 (90.5)	18 (90.0)	0.444
FACIT scale	40.1 ± 13.1	40.7 ± 12.0	39.9 ± 11.2	40.0 ± 12.7	0.978
Barthel scale	92.5 ± 17.4	98.6 ± 4.5	89.6 ± 21.1	95.3 ± 10.6	0.279

Data are expressed as mean ± standard deviations or percentages. Groups were divided according to GLIM (normonutrition, moderate malnutrition, severe malnutrition). Asterisk indicates a significant difference between groups, according to ANOVA (or Kruskal–Wallis *U*-test) and Tukey's multiple comparison test when required (^*^p < 0.05).

^a^Statistically significant differences between normonutrition and moderate malnutrition.

^b^Statistically significant differences between normonutrition and severe malnutrition.

^c^Statistically significant differences between moderate malnutrition and severe malnutrition. Chi-squared test (or Fisher's exact test) was used for variables expressed as percentages (^*^p < 0.05).

BMI, body mass index; FM, fat mass; SGA, subjective global assessment.

**Table 3:** Phenotypic GLIM criteria distribution in malnourished patients according to sex.

	**Malnourished patients**	**Male**	**Female**
	***N** = **62***	***N** = **49***	***N** = **13***
**Involuntary weight loss (%)**			
Moderate (5–10% within the past 6 months) (%)	15 (25.4)	9 (18.3)	6 (46.1)
Severe (>10% within the past 6 months) (%)	39 (62.9)	33 (67.3)	6 (46.1)
**Low body mass index (%)**			
Moderate (< 20 if < 70 years; < 22 if ≥70 years) (%)	3 (4.8)	2 (4.1)	1 (7.7)
Severe (< 18.5 if < 70 years; < 20 if ≥70 years) (%)	0 (0.0)	0 (0.0)	0 (0.0)
**Reduced muscle mass (%)**			
FFMI: < 17 kg/m^2^ (male), < 15 kg/m^2^ (female)	5 (8.1)	4 (8.2)	1 (7.7)
ASMM: < 20 kg (male), < 15 kg (female)	15 (24.2)	12 (24.5)	3 (23.1)
ASMMI: < 7 kg/m^2^ (male), < 5.5 kg/m^2^ (female)	11 (17.7)	10 (20.4)	1 (7.7)

Data are expressed as mean ± standard deviations or percentages. Groups were divided according to sex.

FFMI, fat-free mass index; ASMM, appendicular skeletal muscle mass; ASMMI, appendicular skeletal muscle mass index.

### 3.2 BIVA analysis

Median PhA in SGA-B post-critical COVID-19 patients was 5.40 ± 1.2° and in SGA-C was 4.49 ± 0.8 (*p* < 0.001). SPhA in the SGA-B group was −0.64 ± 1.0 and in the SGA-C group was −1.44 ± 1.0 (*p* = 0.001). BCM in the SGA-B group was 32.0 ± 9.2 kg and in the SGA-C group was 25.11 ± 5.9 kg (*p* < 0.001). BCM/h in the SGA-B group was 18.7 ± 4.8 kg and in the SGA-C group was 14.5 ± 3.0 kg (*p* < 0.001). These data and the rest of the BIVA parameters are shown in Table 4.

**Table 4:** Bioelectrical impedance variables according to SGA.

	**Total**	**SGA-B**	**SGA-C**	***p-*value**
PhA (°)	4.9 ± 1.12	5.40 ± 1.2	4.49 ± 0.8	< 0.001^*^
SPhA	−1.00 (1.1)	−0.64 ± 1.0	−1.44 ± 1.0	0.001^*^
TBW (L)	45.6 ± 9.7	47.4 ± 9.8	41.4 ± 9.1	0.033^*^
ECW (L)	23.3 ± 4.9	23.4 ± 4.6	23.3 ± 5.2	0.064
FM (kg)	30.6 ± 13.4	33.45 ± 15.3	27.1 ± 9.6	0.075
FFM (kg)	59.9 ± 11.7	63.2 ± 12.1	55.89 ± 9.9	0.006^*^
FFMI (kg/m^2^)	20.5 ± 2.9	21.6 ± 2.8	19.1 ± 2.4	< 0.001^*^
BCM (kg)	28.9 ± 8.6	32.0 ± 9.2	25.11 ± 5.9	< 0.001^*^
BCM/h (kg/m)	16.9 ± 4.6	18.7 ± 4.8	14.5 ± 3.0	< 0.001^*^
Na/K exchange	1.15 ± 0.2	1.1 ± 0.2	1.24 ± 0.2	< 0.001^*^
TBW/FFM (%)	76.1 ± 3.9	75.6 ± 3.6	76.6 ± 4.4	0.301
ASMM (kg)	23.6 ± 5.9	25.2 ± 6.0	21.7 ± 5.3	0.013^*^
SMM (kg)	28.8 ± 6.9	30.3 ± 6.8	27.1 ± 6.8	0.046^*^
SMI (kg/m^2^)	9.84 ± 1.8	10.4 ± 1.8	9.2 ± 1.8	0.005^*^
ASMMI (kg/m^2^)	8.07 ± 1.56	8.6 ± 1.6	7.4 ± 1.3	< 0.001^*^

Data are expressed as mean ± standard deviations. Groups were divided according to SGA. Asterisk indicates a significant difference between groups, according to Student's *t*-test (or Mann–Whitney test) (^*^p < 0.05).

PhA, phase angle; SPhA, standardized phase angle; TBW, total body water; ECW, extracellular water; FFM, fat-free mass; BCM, body cell mass; BCM/h, standardized body cell mass; BMI, body mass index; FM, fat mass; ASMM, appendicular skeletal muscle mass; SMI, skeletal mass index; ASMMI, appendicular skeletal muscle mass index; CRP, C-reactive protein; FM, fat mass; HGS, handgrip strength; SMI, skeletal muscle index.

According to GLIM, in normonourished patients, PhA was 5.55 ± 1.2°; in moderately malnourished, PhA was 4.9 ± 0.9°; and in severely malnourished, PhA was 4.8 ± 1.1° (*p* = 0.020). SPhA was −0.397 ± 1.1 in normonourished, −1.4 ± 1.0 in moderate malnourished, and 4.8° ± 1.1 in severe malnourished. BCM was 33.2 ± 8.4 kg in normonourished, 27.7 ± 8.3 kg in moderately malnourished, and 28.2 ± 8.7 kg in severely malnourished, while BCM/h was 19.4 ± 4.4 kg/m in normonourished, 16.4 ± 4.7 kg/m in moderately malnourished, and 16.4 ± 4.8 kg/m in severely malnourished. Other BIVA variables according to GLIM are shown in Supplementary Table 2.

Finally, the representative RzXc graph of our cohort is depicted in Figure 2.

### 3.3 Ultrasound evaluation of RF muscle and abdominal adipose tissue

RF-CSA in post-critical COVID-19 patients was 4.62 ± 1.69 cm^2^ for SGA-B and 3.69 ± 1.0 m^2^ for SGA-C (*p* = 0.008). When standardized for height (RF-CSA/h), we obtained a value of 2.68 ± 0.9 cm^2^/m for SGA-B and 2.11 ± 0.6 cm^2^/m for SGA-C (*p* = 0.007). There were no statistically significant differences in RF-CSA/w.

Muscle thickness (or RF-Y axis) was 1.44 ± 0.5 cm in SGA-B and 1.15 ± 0.3 cm in SGA-C (*p* = 0.008). There were no differences on the RF-X axis. The rest of the ultrasound variables according to SGA are shown in Table 5. NU^®^ variables according to GLIM and their differences are also described (Supplementary Table 3).

**Table 5:** Nutritional ultrasound^®^ variables according to SGA.

	**Total**	**SGA-B**	**SGA-C**	***p-*value**
RF-CSA (cm^2^)	4.21 ± 1.5	4.62 ± 1.69	3.69 ± 1.0	0.008^*^
RF-CSA/h (cm^2^/m)	2.46 ± 0.8	2.68 ± 0.9	2.11 ± 0.6	0.007^*^
RF-CSA/w (cm^2^/Kg)	4.65 ± 1.35	4.87 ± 1.43	4.33 ± 1.16	0.119
RF-CIR	9.0 ± 1.4	9.27 ± 1.5	8,62 ± 1.1	0.075
RF*-X* axis (cm)	3.68 ± 0.6	3.73 ± 0.7	3.73 ± 0.5	0.933
RF-Y axis (cm)	1.32 ± 0.4	1.44 ± 0.5	1.15 ± 0.3	0.008^*^
L-SAT (cm)	1.04 ± 0.6	1.08 ± 0.7	0.98 ± 0.6	0.767
T-SAT (cm)	1.89 ± 0.9	2.04 ± 1.0	1.64 ± 0.6	0.088
VAT (cm)	0.73 ± 0.5	0.80 ± 0.5	0,60 ± 0.3	0.145
VAT/h (cm/m)	0.427 ± 0.3	0.47 ± 0.3	0.35 ± 0.2	0.148
VAT/w (cm/kg)	0.008 ± 0.0	0.008 ± 0.0	0.007 ± 0.0	0.489

Data are expressed as mean ± standard deviations. Groups were divided according to SGA. Asterisk indicates a significant difference between groups, according to Student's *t*-test (or Mann–Whitney test) (^*^p < 0.05).

RF-CSA, rectus femoris cross-sectional area; RF-CSA/h, cross-sectional area/height; RF-CIR, circumference of quadriceps rectus femoris; L-SAT, leg subcutaneous adipose tissue; T-SAT, total subcutaneous abdominal adipose tissue; VAT, total visceral adipose tissue; VAT/h, total visceral adipose tissue/height; VAT/w, total visceral adipose tissue/weight; SGA, subjective global assessment.

### 3.4 Functional status assessment

Overall mean right handgrip strength was 26.5 ± 13.8 kg for SGA-B and 22.1 ± 9.4 kg for SGA-C (*p* = 0.183), whereas left handgrip strength was 24.7 ± 13.8 kg for SGA-B and 19.6 ± 10.6 kg for SGA-C (*p* = 0.147).

No statistically significant differences between other functional parameters were detected between SGA-B and SGA-C (Table 6).

**Table 6:** Functional test variables according to SGA.

	**Total**	**SGA-B**	**SGA-C**	***p-*value**
R-HGS (kg)	24.7 ± 12.3	26.5 ± 13.8	22.1 ± 9.4	0.183
L-HGS (kg)	22.5 ± 12.7	24.7 ± 13.8	19.6 ± 10.6	0.147
UAG (s)	8.49 ± 2.7	8.74 ± 2.7	8.17 ± 2.9	0.675
>12 s	2 (14.3)	1 (10.0)	1 (12.5)	
>20 s	0 (0.0)	0 (0.0)	0 (0.0)	
6 MWT (m)	369 ± 107	347 ± 111	384 ± 102	0.276
>350 m	30 (58.8)	15 (60.0)	15 (57.7)	
< 350 m	21 (41.2)	10 (40.0	11 (42.3)	

Data are expressed as mean ± standard deviations or percentages. Groups were divided according to SGA. Asterisk indicates a significant difference between groups, according to Student's *t*-test (or Mann–Whitney test) (^*^p < 0.05). Chi-squared test (or Fisher's exact test) was used for variables expressed as percentages (^*^p < 0.05).

R-HGS, right handgrip strength; L-HGS, left handgrip strength; TUG, timed get-up-and-go; 6 MWT, 6-min walk test. SGA, subjective global assessment.

### 3.5 Biochemical analysis

Prealbumin tends to be higher in SGA-B (28.2 ± 14.1 mg/dL) with respect to SGA-C (25.4 ± 7.9 mg/dL), while with CRP, the opposite occurs, the prealbumin is lower in SGA-B (21.3 ± 27.1 mg/L) with respect to SGA-C (33.9 ± 38.3 mg/L), in both cases without reaching statistical significance (*p* = 0.642 and *p* = 0.818, respectively). In addition, there are also no differences between vitamin D levels. These data are described in Supplementary Table 4.

### 3.6 Correlation of new NU^®^ values with validated measurement of BIVA parameters, functional status assessment, and complications in post-critical COVID-19 patients

RF-CSA correlated negatively with the total hospital stay (*r* = −0.22, *p* < 0.001) and the need for IMV (*r* = −0.28, *p* = 0.028). On the other hand, RF-CSA correlated positively with a greater BCM/h (*r* = 0.41, *p* < 0.001), SMI (*r* = 0.58, *p* < 0.001), RF-Y axis (*r* = 0.69, *p* < 0.001), and HGS assessed using dynamometry (*r* = 0.50, *p* < 0.001) and the Barthel scale (*r* = 0.15, *p* = 0.005). It did not correlate with days of IMV or the need for tracheostomy.

**Figure 3:**
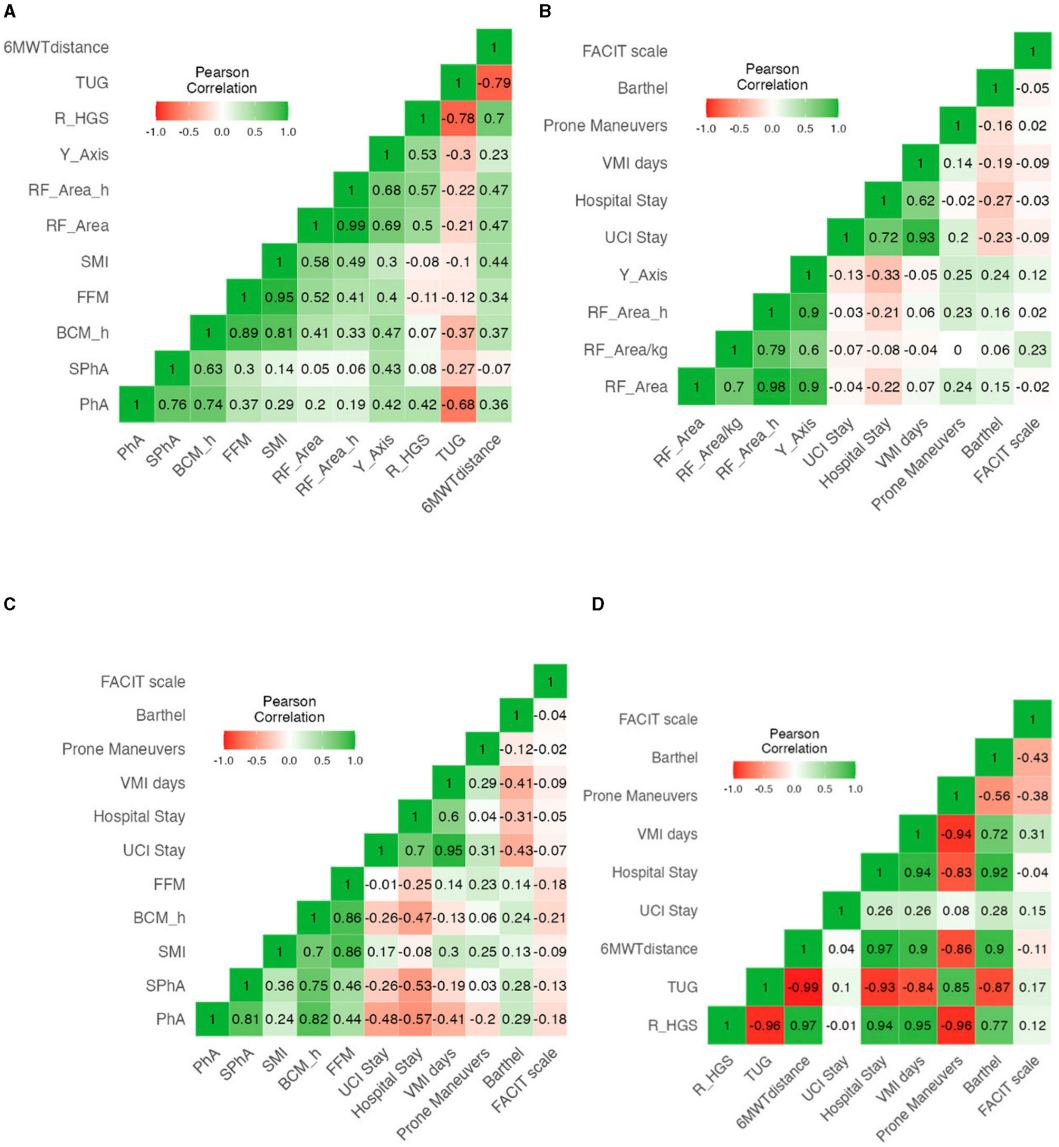
**(A)** Correlation heatmap of BIVA variables, Nutritional Ultrasound parameters, and functional test in post-critical COVID-19 outpatients. **(B)** Correlation heatmap of NU^®^ parameters with complications and aggressive therapy requirements in post-critical COVID-19 outpatients. **(C)** Correlation heatmap of BIVA parameters with complications and aggressive therapy requirements in post-critical COVID-19 outpatients. **(D)** Correlation heatmap of functional test with complications and aggressive therapy requirements in post-critical COVID-19 outpatients.

PhA is a global cellular health parameter that measures the nutritional and inflammatory status of patients. It was negatively correlated with ICU stay (*r* = −0.48, *p* < 0.001), total hospital stay (*r* = −0.57, *p* < 0.001), need for invasive mechanical ventilation (*r* = −0.39, *p* < 0. 001), days of invasive mechanical ventilation (*r* = −0.41, *p* = 0.003), need for tracheostomy (*r* = −0.507, *p* < 0.001), and number of prone maneuvers (*r* = −0.20, *p* < 0.001). On the other hand, it was positively correlated with BCM/h (*r* = 0.74, *p* < 0.001), SMI (*r* = 0.29, *p* < 0.001), RF-CSA (*r* = 0.22, *p* < 0.001), and RF-Y axis (*r* = 0.42, *p* < 0.001). HGS was assessed using dynamometry (*r* = 0.42, *p* < 0.001) and the Barthel scale (*r* = 0.29, *p* = 0.005). These data and further correlations are shown in Figure 3, and their respective *p*-values are shown in Supplementary Tables 5–7.

### 3.7 Performance of BIVA and NU^®^ to predict severe malnutrition

#### 3.7.1 BIVA parameters

**Figure 4:**
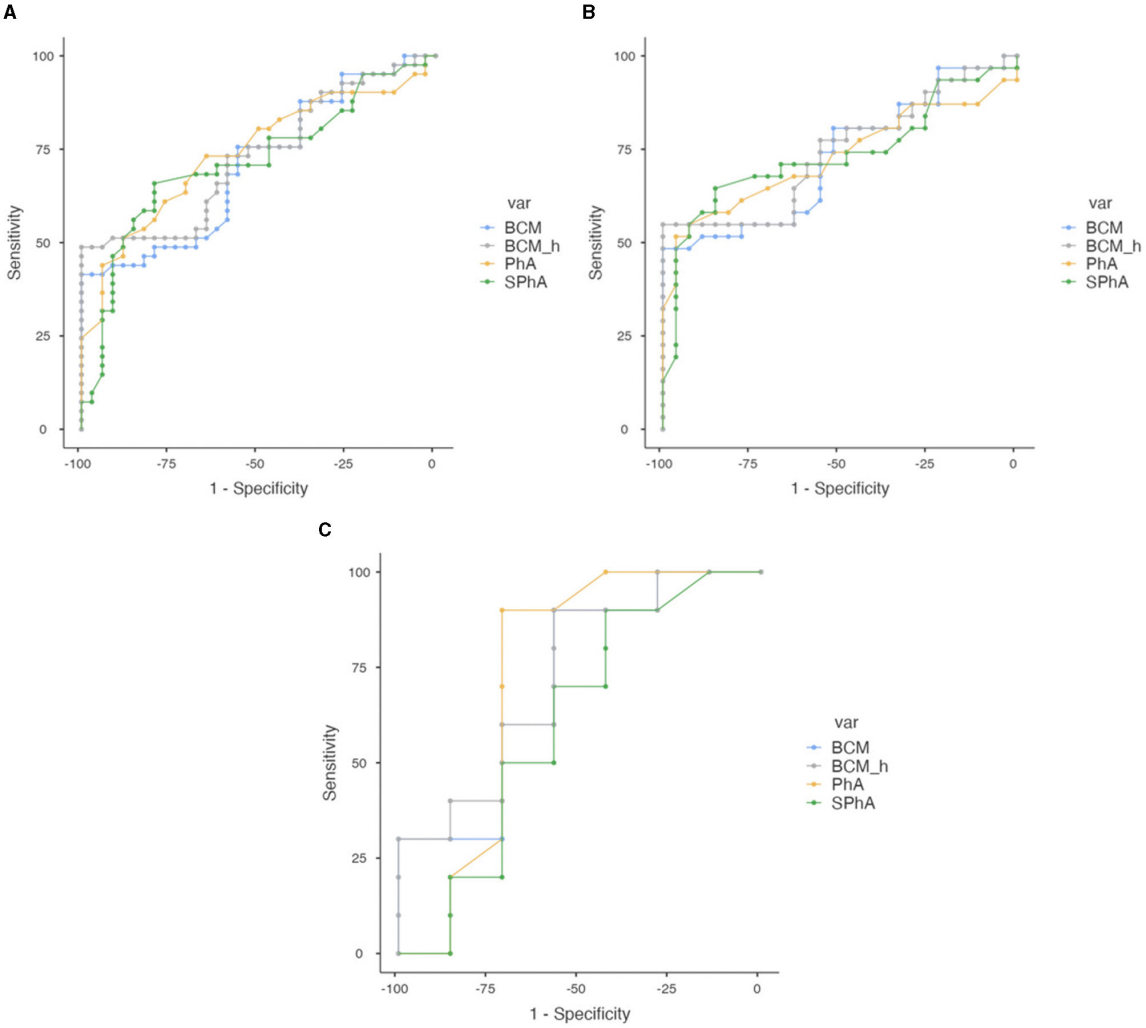
Combined ROC curve analysis of PhA, SPhA, BCM, and BCM/h to assess severe malnutrition according to SGA in overall COVID-19 post-critical outpatients. **(A)** Total sample. **(B)** Men. **(C)** Women.

To examine the capacity of BIVA parameters to predict severe malnutrition according to SGA in our population, we performed an ROC analysis. With PhA, the AUC was 0.740 and the optimal cutoff point for maximum efficacy was 5.4°, with sensitivity (SE) of 51.22%, specificity (SP) of 88.24%, positive predictive value (PPV) of 84%, and negative predictive value (NPV) of 60%. In the men's group, the AUC was 0.745 with an optimal cutoff point of 5.7, and for women, the AUC was 0.737 with an optimal cutoff point of 4.8. According to SPhA, the AUC was 0.717 with an optimal cutoff point for maximum efficacy of −0.79 (SE = 79.41%, SP = 79.41%, PPV 79.41%, and NPV 65.85%). With BCM, the AUC was 0.707 with an optimal cutoff point for maximum efficacy of 34.5 kg (SE 41.46%, SP 100%, PPV 100%, and NPV 58.62%), while for men the AUC was 0.726 with an optimal cutoff point of 34.90 kg (SE 48.39%, SP 100%, VPP 100%, and VPN 62.79%), and for women, the AUC was 0.714 with a cutoff point of 23.5 kg. With BCM/h, the AUC was 0.730 with an optimal cutoff point for maximum efficacy of 18.36 kg/m, for men, the AUC was 0.741 with a cutoff point of 19.27 kg/m, and for women, the AUC was 0.729 with an optimal cutoff point of 14.00 kg/m, as shown in Figure 4.

In addition, ROC curves are performed for FFMI, ASMM, and ASMMI, which are the most commonly used parameters of reduced muscle mass in international criteria such as GLIM, to find the cutoff points that best discriminate severe malnutrition from moderate malnutrition according to SGA in our cohort. While data are missing for women, it is possible for men. Using FFMI, the AUC was 0.749 with an optimal cutoff point at 21.37 kg/cm^2^ (SE 58.54%, SP 88.24%, PPV 85.71%, and NPV 63.83%), while for men, the AUC was 0.769 with an optimal cutoff point also at 21.37 kg/cm^2^ (SE 61.29%, SP 85.19%, PPV 82.61%, and NPV 65.71%). For ASMM, the AUC was 0.668 with the optimal cutoff point at 26.7 kg (SE 41.46%, SP 88.24%, PPV 80.95%, and NPV 55.56%), and for men, the AUC was 0.661 with the optimal cutoff point at 26.7 kg (SE 45.16%, SP 85.19%, PPV 77.68%, and NPV 57.59%). For ASMMI, the AUC was 0.713 with the optimal cutoff point at 8.56 kg/cm^2^ (SE 53.66%, SP 82.35%, PPV 78.57%, and NPV 59.57%), while for men, the AUC was 0.704 with the optimal cutoff point at 8.46 kg/cm^2^ (SE 58.06%, SP 77.78%, PPV 75%, and NPV 61.76%), as shown in Supplementary Figure 1.

#### 3.7.2 Nutritional ultrasound^®^ parameters

**Figure 5:**
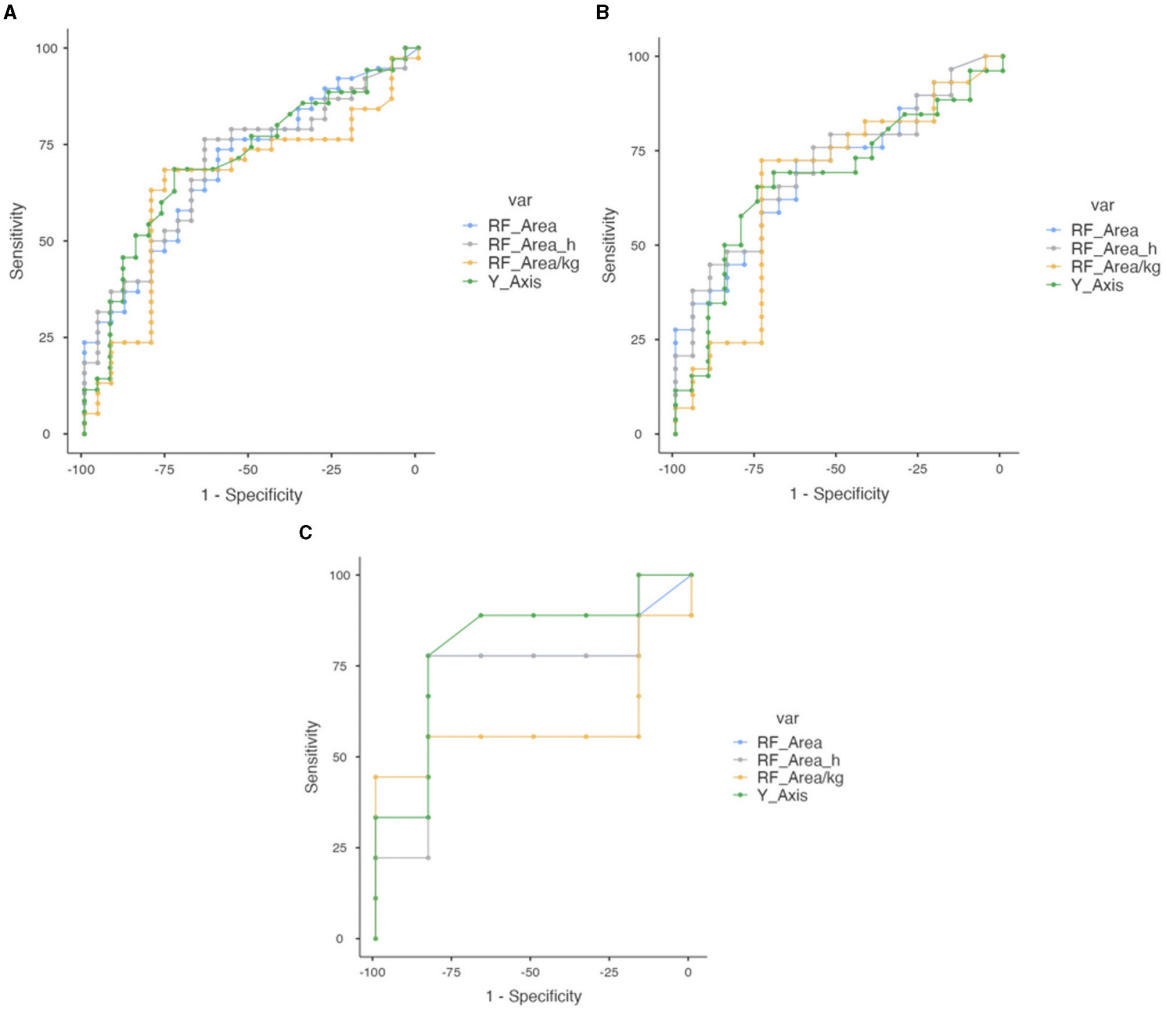
Combined ROC curve analysis of RF-CSA, RF-CS/height, RF-CSA/weight, and RF-Y axis to assess severe malnutrition in overall COVID-19 post-critical outpatients. **(A)** Total sample. **(B)** Men. **(C)** Women.

Using RF-CSA, the AUC was 0.693 and the optimal cutoff point was 3.62 cm^2^ (SE 73.68%, SP 60%, PPV 73.68%, and NPV 60%), for men, the AUC was 0.701 with the optimal cutoff point at 3.80 cm^2^ (SE 72.41%, SP 63.16%, PPV 75%, and NPV 60%), and for women, the AUC was 0.731 with the optimal cutoff point at 3.53 cm^2^ (SE 77.781%, SP 83.33%, PPV 87.5%, and NPV 71.43%). When we standardized by height (RF-CSA/h), the AUC improves to 0.697, with an optimal cutoff point for maximum efficacy of 2.19 cm^2^/m (SE 76.32%, SP 64%, PPV 76.32%, and NPV 64%), for men, the AUC was 0.708 with the optimal cutoff point at 2.52 cm^2^/m (SE 62.07%, SP 73.68%, PPV 78.26%, and NPV 56%), and for women, the AUC was 0.722 with the optimal cutoff point at 2.21 cm^2^/m (SE 77.78%, SP 83.33%, PPV 87.5%, and NPV 71.43%). With the RF-Y axis, the global AUC was 0.706 with the optimal cutoff point at 1.21 cm (SE 68.57%, SP 73.08%, PPV 77.42%, and NPV 58.82%), for men, the AUC was 0.678 with an optimal cutoff point of 1.25, and for women, the AUC was 0.806 with an optimal cutoff point of 1.17. These data are shown in Figure 5.

#### 3.7.3 Functional tests

Functional tests did not show good performance in creating cutoff points to assess severe malnutrition expressed as AUC. Using dynamometry, the AUC was 0.548 with an optimal cutoff point at 29 kg. For men, the AUC was 0.568 with an optimal cutoff point at 40 kg. For women, the AUC was 0.611 with the optimal cutoff point at 10 kg. These results are shown in Supplementary Figure 2.

#### 3.7.4 Factors involved in severe malnutrition. Multivariate logistic regression analysis

To evaluate factors associated with severe malnutrition characterized by SGA-C, a multivariate logistic regression analysis was performed (Table 7), considering as the dependent variable the nutritional status according to SGA. The optimum model that best explained severe malnutrition included PhA (*p* = 0.014), RF-CSA/h (*p* = 0.048), total hospital stay (*p* = 0.034), and ICU stay (*p* = 0.009).

**Table 7:** Multivariate regression analysis of predictors of severe malnutrition according to SGA.

**Predictor**	**95% confidence interval**				
	**B**	***p*-value**	**Odds ratio**	**Lower**	**Upper**
PhA	−1.1308	0.014	0.323	0.131	0.794
ICU stay (days)	0.0799	0.009	1.083	1.020	1.150
Hospital stay (days)	−0.0264	0.034	0.974	0.950	0.998
RF-CSA/h (cm^2^/m)	−0.7288	0.048	0.582	0.268	0.993

Logistic regression analysis: risk (odds ratio) of severe malnutrition. Statistical significance at *p* < 0.05. Dependent variable: nutritional status according to SGA.

PhA, phase angle; RF-CSA/h, cross-sectional area/height; ICU, intensive care unit.

### 3.8 Performance of the cutoff points previously described for assessing complications and need for aggressive therapies during admission

To determine whether the cutoff points found in the previous section would be clinically useful, we analyzed the differences between the two groups above and below the cutoff point, in terms of complications and aggressive therapy requirements during admission. We found statistically significant differences when we set a PhA < 5.4° in ICU stay (*p* = 0.005), total hospital stay (*p* < 0.001), number of prone maneuvers (*p* = 0.002), need for IMV (*p* = 0.002), and need for rehabilitation (*p* = 0.022). Setting an SPhA < −0.79, we also found differences in ICU stay (*p* = 0.003), hospital stay (*p* < 0.001), number of prone maneuvers (*p* = 0.012), need for IMV (*p* = 0.005), and need for rehabilitation (*p* = 0.004), even with a lower *p*-value.

In contrast, we did not find statistically significant differences when using global RF-CSA, RF-CSA/h, and HGS in our cohort. However, when divided by sex, we did find differences. Thus, using an RF-CSA < 3.80 cm^2^ in men, we observed differences in hospital stay (*p* = 0.009), while using an RF-CSA/h < 2.52 cm^2^/m, we found differences in ICU stay (*p* = 0.049) and hospital stay (*p* = 0.010). In the analysis of women, we found statistically significant differences only when using an RF-CSA/h < 2.21 cm^2^/m in hospital stay (*p* = 0.022) and Barthel scale (*p* = 0.039). These results are shown in Table 8.

**Table 8:** Complications and aggressive therapy requirements according to PhA, SPhA, RF-CSA, and RF-CSA/h cutoff points for severe malnutrition.

	**PhA < 5.4 (*n* = 50) vs. PhA ≥5.4 (*n* = 25) (*p*-value)**	**SPhA < −0.79 (*n* = 40) vs. SPhA ≥-0.79 (*n* = 35) (*p*-value)**	**RF-CSA < 3.80 (*n* = 20) vs. RF-CSA ≥3.80 (*n* = 38) (cm^2^) (men) *(p*-value)**	**RF-CSA < 3.53 (*n* = 7) vs. RF-CSA ≥3.53 (*n* = 10) (cm^2^) (women) *(p*-value)**	**RF-CSA/h < 2.52 (*n* = 26) vs. RF-CSA/h ≥2.52 (*n* = 32) (cm^2^/m) (men) *(p*-value)**	**RF-CSA/h < 2.21 (*n* = 7) vs. RF-CSA/h ≥2.21 (*n* = 10) (cm^2^/m) (women) *(p*-value)**
ICU stay (days)	26.25 vs. 14.5 (*p* = 0.005)	28.07 vs. 15.76 (*p* = 0.003^*^)	29.52 vs. 23.41 (*p* = 0.067)	17.12 vs. 11.88 (*p* = 0.601)	29.38 vs. 22.56 (*p* = 0.049^*^)	15.00 vs. 10.80 (*p* = 0.590)
Hospital stay (days)	56.8 vs. 29.0 (*p* = < 0.01^*^)	61.88 vs. 30.91 (*p* < 0.01^*^)	72.05 vs. 41.70 (*p* = 0.009^*^)	45.12 vs. 27.12 (*p* = 0.961)	68.00 vs. 40.25 (*p* = 0.010^*^)	47.29 vs. 23.20 (*p* = 0.494)
IMV (%)	83.72 vs. 50.0 (0.002^*^)	69.7 vs. 37.50 (*p* = 0.005^*^)	76.19 vs. 51.35 (*p* = 0.063)	50.0 vs. 44.44 (*p* = 0.819)	69.23 vs. 53.12 (*p* = 0.212)	57.14 vs. 40.0 (*p* = 0.486)
IMV (days)	23.0 vs. 28.7 (*p* = 0.307)	30.10 vs. 22.31 (0.213)	30.25 vs. 32.37 (*p* = 0.868)	11.38 vs. 11.18 (*p* = 1.0)	21.83 vs. 30.94 (*p* = 0.882)	11.25 vs. 12.25 (*p* = 1.0)
Home oxygen therapy after discharge (%)	21.0 vs. 12.0 (*p* = 0.622)	16.0 vs. 17.0 (*p* = 0.456)	45.0 vs. 42.1 (*p* = 0.832)	42.9 vs. 50.0 (*p* = 772)	46.2 vs. 40.6 (*p* = 0.672)	42.9 vs. 50.0 (*p* = 0.772)
Rehabilitation (%)	85.29 vs. 57.57 (*p* = 0.022^*^)	76.47 vs. 39.39 (*p* = 0.005^*^)	57.14 vs. 35.13 (*p* = 0.251)	62.5 vs. 44.44 (*p* = 0.607)	50.0 vs. 37.5 (*p* = 0.600)	57.14 vs. 50.0 (*p* = 0.880)
Maneuvers prone (*n*)	2.94 vs. 1.22 (*p* = 0.002^*^)	3.00 vs. 1.67 (*p* = 0.012^*^)	3.25 vs. 2.41 (*p* = 0.131)	1.61 vs. 1.33 (*p* = 1.0)	3.08 vs. 2.41 (*p* = 0.187)	1.57 vs. 1.30 (*p* = 0.710)
Barthel scale	90.4 vs. 97.8 (*p* = 0.066)	90.48 vs. 95.54 (*p* = 0.093)	86.43 vs. 94.86 (*p* = 0.061)	91.12 vs. 100.0 (*p* = 0.244)	89.04 vs. 94.00 (*p* = 0.322)	91.43 vs. 100.0 (*p* = 0.273)
FACIT Fatigue scale	41.12 vs. 37.7 (*p* = 0.473)	40.83 vs. 39.12 (*p* = 0.869)	40.57 vs. 42.17 (*p* = 0.235)	40.15 vs. 33.50 (*p* = 0.699)	40.15 vs. 42.77 (*p* = 0.193)	41.43 vs. 30.50 (*p* = 0.260)

Data are expressed as mean (quantitative variables) or percentage (qualitative variables). Groups were divided according to the value above and below the cutoff points for assessing severe malnutrition. Asterisk indicates a significant difference between groups, according to Student's *t*-test (or Mann–Whitney test) (^*^p < 0.05). Chi-squared test (or Fisher's exact test) was used for variables expressed as percentages (^*^p < 0.05).

PhA, phase angle; SPhA, standardized phase angle; FFMI, fat-free mass index; ASMMI, appendicular skeletal muscle mass index; BCM, body cell mass; BCM/h, standardized body cell mass; ICU, intensive care unit; IMV, invasive mechanical ventilation.

## 4 Discussion

The main finding of the study is that PhA and RF-CSA show good performance in predicting severe malnutrition according to SGA and assessing the presence of complications and aggressive therapy requirements in post-critical COVID-19 patients.

The diagnosis of disease-related malnutrition is not an easy task, especially in obese population. In fact, our patient sample is representative of the COVID-19 population at risk of severe illness and critical care, as it has a high percentage of obese patients (61.8% according to BMI > 30; 48.0% according to FM > 30% in men and >40% in women), which is associated with a longer ICU stay in the literature, as obesity is a risk factor for hospitalization and severity of illness (19, 38). Muscle involvement component is more difficult to assess due to the high BMI of these patients, therefore malnutrition may be hidden, which is why a complete morphofunctional assessment may be useful in this population.

There is no global consensus on the approach to assessing malnutrition; there are many parameters that can be used, each with its advantages and limitations. Some parameters, such as weight loss, BMI, muscle mass, or food intake, are included in most malnutrition screening tools, while others, such as functional parameters and QoL, have gradually gained protagonism (37).

The SGA classification (26, 27) and the GLIM criteria (22, 27) are the two most currently used tools for the diagnosis of malnutrition. In our study, statistically significant differences in most of the parameters of morphofunctional assessment were seen between most of the SGA and GLIM subcategories.

However, in the case of GLIM criteria, although differences are significant between normonourished and malnourished patients, there are no differences between moderate malnutrition vs. severe malnutrition subcategories. The explanation could be that these two subcategories are currently only defined by percentage weight loss and low body mass index, without taking into account muscle mass loss due to the lack of cutoff points. Applied to our study, this subclassification relies in most cases on the percentage of weight loss as the determining factor (which classifies 25.5% of patients as moderately malnourished and 66.1% as severely malnourished). Since the average BMI is 31.1 ± 6.4 kg/m^2^, a low body mass index only classifies 5.0% of patients as moderately malnourished and 0% as severely malnourished (43–45). GLIM criteria still fail to discriminate the severity of muscle mass loss as more studies are needed to define robust age- and sex-specific cutoff points (46).

Current GLIM criteria guideline (47) recommends the assessment of FFM and other muscle mass parameters such as ASMM and ASMMI using BIA as diagnostic criteria for malnutrition and sarcopenia, although without determining specific cutoff points for classification as moderate or severe malnutrition. For the purposes of recommended cutoff values for muscle mass reductions, GLIM refers to the recommendations of the European Working Group on Sarcopenia in Older People 2 (EWGSOP2) (48), the National Institute of Health Foundation (NIHF) initiative (49), and the Asian Working Group on Sarcopenia (AWGS) (50). Further research is needed to establish general reference standards adjusted for race, age, and sex. Examples of recommended thresholds for malnutrition are a FFMI < 17 kg/m^2^ for men and < 15 kg/m^2^ for women (51, 52). In addition, following the EWGSOP2 and its definition of sarcopenia, other thresholds are an ASMMI < 7 kg/m^2^ for men and < 5.5 kg/m^2^ for women, an ASMM < 20 kg for men and < 15 kg for women, or a reduction in muscle strength (< 27 kg for men and < 16 kg for women), although HGS has not yet been accepted as a phenotypic GLIM criterion but only as a supportive measure (47, 48).

Following these recommendations, in malnourished patient subgroup according to GLIM criteria, only 8.4% of patients were observed with low FFMI, while 24.2% had low ASMM (contributing a total of 10 more patients with respect to FFMI; 16.1% of malnourished patients) and 17.7% with low ASMMI (6 more patients; 9.7%), as shown in Table 3. These percentages according to muscle mass parameters could be more convincing if in the future cutoff points are found based on a younger population than those provided by the EWGSOP2 (48) and Cederholm et al. (52).

In addition, despite being a subjective classification, SGA includes a wider range of clinical characteristics that support it and is still the gold standard in the assessment of disease-related malnutrition (28). This reason and those mentioned in the preceding paragraph are the reasons why we decided to use SGA vs. GLIM in order to define moderate and severe malnutrition subcategories that are the basis of our study.

Our study sought to assess malnutrition in post-critical COVID-19 outpatients. SGA and GLIM criteria encounter challenges when attempting to accurately assess the body composition or functional status of patients independently. Therefore, a complete morphofunctional assessment that encompasses emerging parameters such as PhA and RF-CSA is needed in addition to other preexisting tools as they have been shown to hold diagnostic and prognostic value in disease-related malnutrition (17, 18) and a positive correlation between each other. Figure 3 shows a statistically significant positive correlation between all parameters related to muscle function and muscle mass, including PhA, BCM/h, RF-CSA, HGS, and Barthel scale, demonstrating high coherence and complementarity of each of the parts of the morphofunctional assessment in our population.

Morphofunctional analysis showed worse results in the SGA-C subgroup in our sample. In the RzXc graph, the vector of the SGA-C subgroup indicates a higher degree of inflammation and a lower cell mass than in the SGA-B subgroup (Figure 2), according to a lower PhA, with a worse prognosis (18).

PhA has already been studied as a prognostic marker in malnutrition, specifically in COVID-19 patients (18, 53). Although there are no published data on post-critical patients, recent studies suggest that PhA may be useful for predicting malnutrition, both (52) according to GLIM criteria and SGA (54–56). In patients admitted to the ICU, measurement of PhA predicted COVID-19 severity and was associated with 90-day mortality, with longer hospital stay and higher inflammation markers (18–20). In our study, PhA showed good performance in predicting severe malnutrition and was associated with a significant increase in ICU stay, hospital stay, number of prone maneuvers, need for IMV, and rehabilitation. With SPhA < −0.79, we also observed these associations, with even lower *p*-values adjusted for age and sex (19).

In a recent study, RF-CSA by NU^®^ provided the possibility of assessing sarcopenia when applied to the assessment of body composition in post-critical COVID-19 patients (7). However, as far as we know, there is no existing literature on the performance of RF-CSA to predict malnutrition. Nevertheless, RF-CSA/h demonstrated usefulness in predicting severe malnutrition and assessing complications within our population.

These findings have important clinical relevance, as they are highly available, inexpensive, safe for the patient, efficient, and fast tests, which can be performed at the bedside multiple times during admission and follow-up. It is likely, in the near future, that we will have more experience with these techniques and more studies will emerge to unify cutoff points for BIVA and NU^®^ measurements as prognostic markers in the critically and post-critically ill patient.

In cases of malnutrition due to acute or subacute pathology, classical parameters such as weight, BMI, albumin, and prealbumin are often not useful. Weight and BMI are rarely below normal, given the short evolution of the disease. Moreover, albumin, being a negative acute phase reactant, is synthesized to a lesser extent by the liver during this acute proinflammatory state, while at the same time generating a greater amount of CRP (57). This is why we must look at other aspects such as percentage weight loss, and more importantly, parameters that reflect muscle mass and cellular health such as PhA, FFM, BCM, or Na/K exchange to determine whether the patient is malnourished and to be able to implement appropriate and timely nutritional support.

To the best of our knowledge, this is the first study combining NU, BIVA, and functional tests to assess severe malnutrition and complications in a post-critical COVID-19 outpatient cohort.

## 5 Limitations

This study has some limitations. This is a single-center study, with a small sample size, where only surviving ICU patients were assessed without taking into account those who died during their ICU stay. Another limitation is the inability to perform anthropometric measurements in critical patients during ICU admission, which limits the spectrum of disease severity in our sample. In addition, given the predominance of men in severe COVID-19 studies, as is the case in our cohort, drawing conclusions by gender in body composition is difficult given the small sample of women.

Unfortunately, a significant number of patients who belonged to other hospitals or were foreign patients returning to their home countries were excluded from the study. In addition, 55 patients were lost to follow-up and not included in the study, likely resulting from the closure of post-COVID follow-up appointments during the third wave in January 2021.

Furthermore, a lack of significant differences in functional tests could be due to the fact that muscle function recovers quickly after starting the nutritional intervention at discharge. Our assessment takes place 14–21 days after hospital discharge so that patients may have already experienced an overall improvement in functional tests, which makes statistical significance difficult and becomes a limitation of our study.

Further prospective and multi-center studies will be needed to define shared cutoff points of muscle mass loss to apply to the definition of malnutrition in critical and post-critical patients.

## 6 Conclusion

Our study shows that more than 75% of the post-critical COVID-19 survivors had malnutrition, and approximately half were obese. PhA, SPhA, RF-CSA, and RF-CSA/h measurements, when applied to the assessment of body composition in post-critical COVID-19 patients, showed a statistically significant positive correlation with other morphofunctional parameters and clinical results and had good performance in predicting severe malnutrition according to SGA and assessing complications and aggressive therapy requirements during admission. It reinforces the implementation of PhA and RF-CSA in routine clinical practice and its use as a potential phenotypic criterion for muscle mass loss into the GLIM criteria so they can improve the assessment of malnutrition in post-critical COVID-19 survivors, although more studies are needed to assess the performance of these methods in other populations.

## Data availability statement

The raw data supporting the conclusions of this article will be made available by the authors, without undue reservation.

## Ethics statement

The studies involving humans were approved by Ethics Committee of Virgen de la Victoria University Hospital (PI-20-321, September 2021). The studies were conducted in accordance with the local legislation and institutional requirements. The participants provided their written informed consent to participate in this study.

## Author contributions

VS-F, IC-P, and JG-A: conceptualization. VS-F, IV-A, AS-G, PM-L, PN-O, CR-A, ME-F, AG-G, MG-J, EA-M, and RF-J: methods. VS-F, RF-J, IC-P, JG-A, and FT-M: formal analysis, writing-review and editing, visualization, and supervision. VS-F and JG-A: writing original draft preparation. All authors have read and agreed to the published version of the manuscript.

## Supplementary material

Supplementary Figure 1 Combined ROC curve analysis of FFMI, ASMM and ASMMI to assess severe malnutrition according to SGA in overall COVID-19 post-critical outpatients. **(A)** Total sample. **(B)** Men. **(C)** Women.

Supplementary Figure 2 ROC curve analysis of HGS to assess severe malnutrition in overall COVID-19 post-critical outpatients. **(A)** Total sample. **(B)** Men. **(C)** Women.

Supplementary Table 1 Baseline characteristics and morphofunctional assessment parameters, according to sex.

Supplementary Table 2 Bioelectrical Impedance variables according to GLIM.

Supplementary Table 3 Nutritional Ultrasound^®^ variables according to GLIM.

Supplementary Table 4 Biochemical variables according to SGA.

Supplementary Table 5 Correlation matrix of BIVA variables, nutritional ultrasound parameters and functional test in post-critical COVID-19 outpatients.

Supplementary Table 6 Correlation matrix of nutritional ultrasound parameters, complications and need for aggressive therapies in post-critical COVID-19 outpatients.

Supplementary Table 7 Correlation matrix of BIVA variables, complications and need for aggressive therapies in post-critical COVID-19 outpatients.
