# Supplementary figures and images for: Phase angle and rectus femoris cross-sectional area as predictors of severe malnutrition and their relationship with complications in outpatients with post-critical SARS-CoV2 disease
Source: Front Nutr. 2023 Nov 21;10:1218266. doi: 10.3389/fnut.2023.1218266 (PMC10702576; doi:10.3389/fnut.2023.1218266)

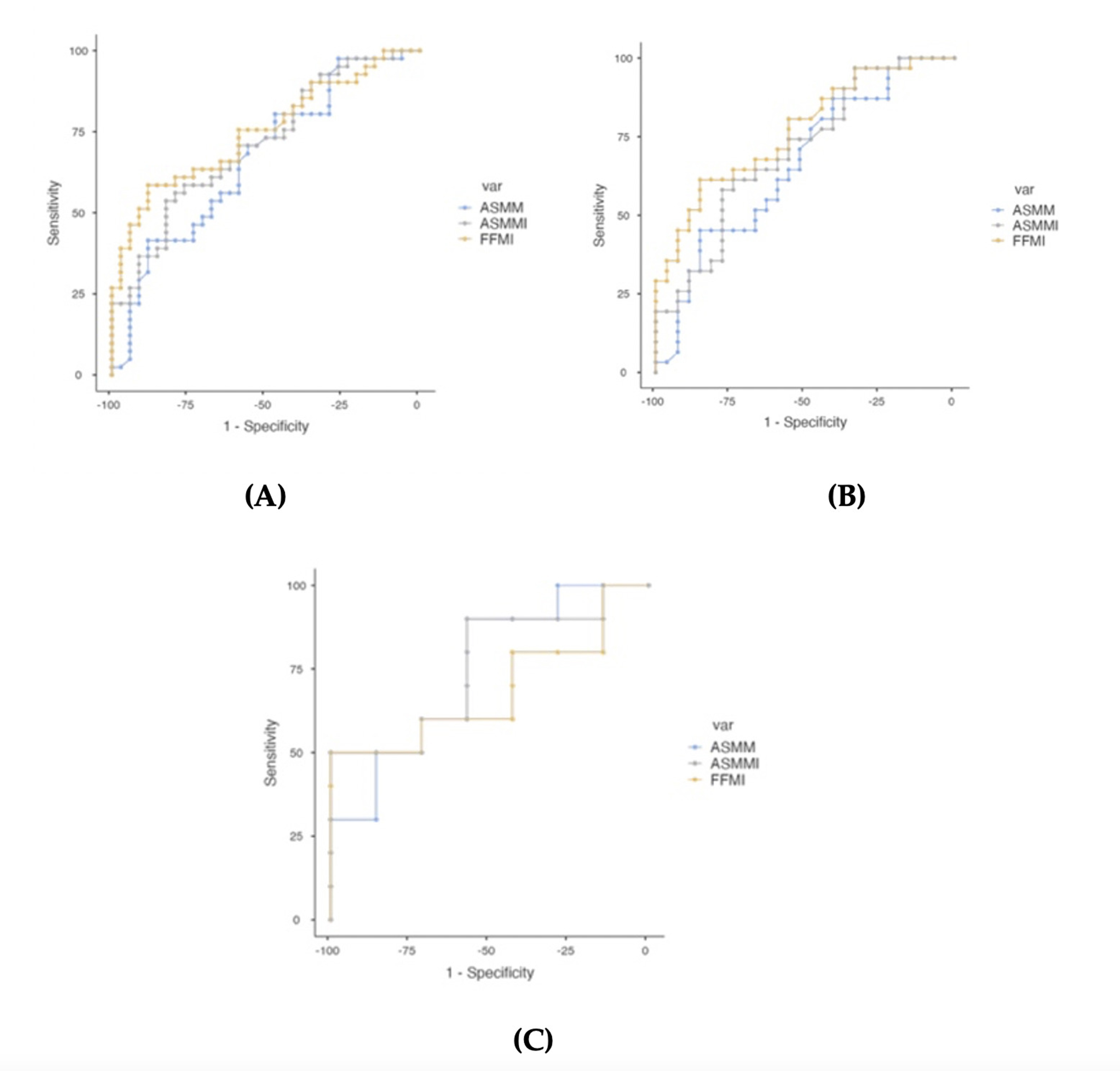

Supplement: Supplementary Figure 1 — Combined ROC curve analysis of FFMI, ASMM and ASMMI to assess severe malnutrition according to SGA in overall COVID-19 post-critical outpatients. (A) Total sample. (B) Men. (C) Women. [file Image_1.JPEG]

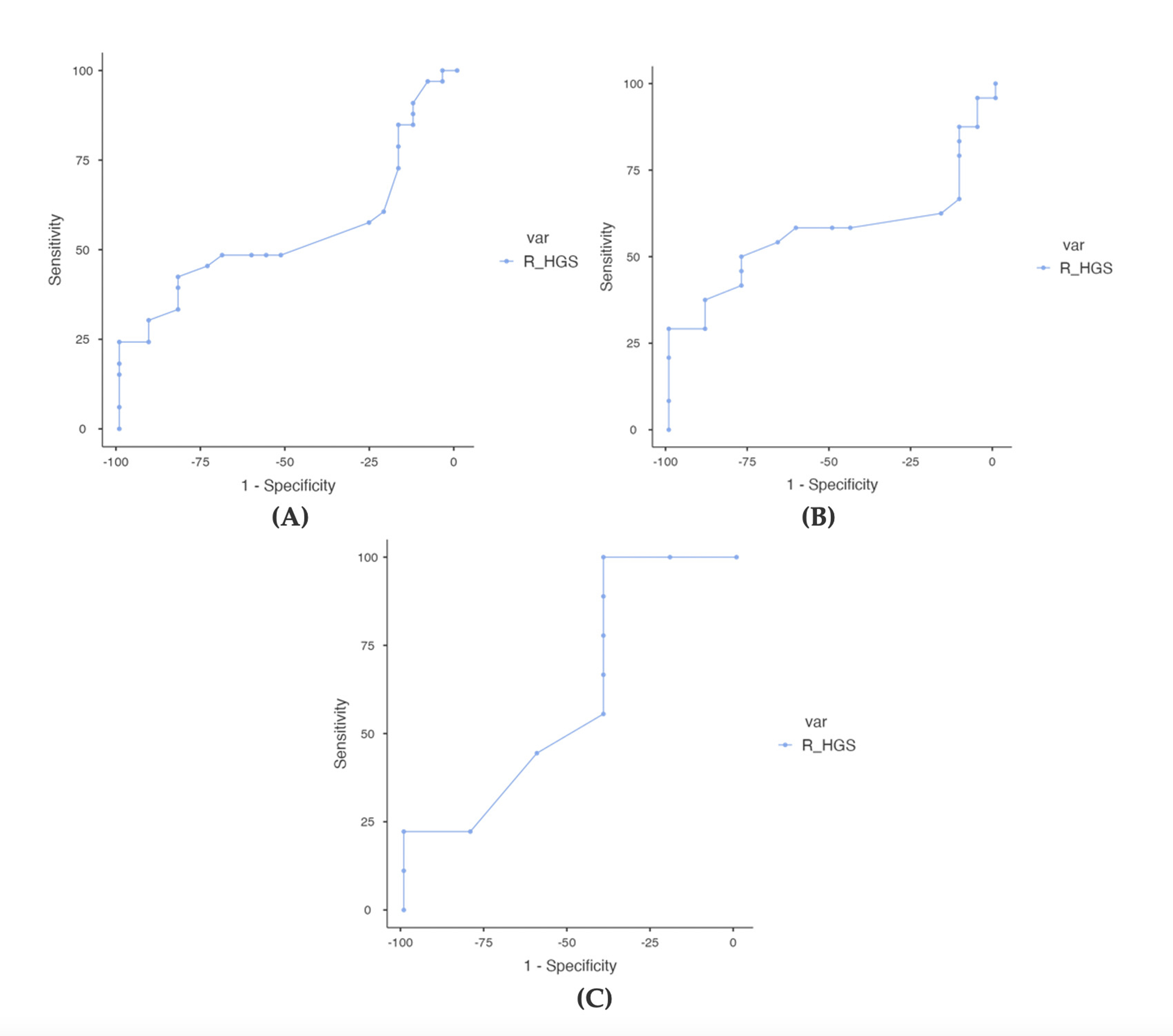

Supplement: Supplementary Figure 2 — ROC curve analysis of HGS to assess severe malnutrition in overall COVID-19 post-critical outpatients. (A) Total sample. (B) Men. (C) Women. [file Image_2.JPEG]
