# Supplementary material for: Phase angle and rectus femoris cross-sectional area as predictors of severe malnutrition and their relationship with complications in outpatients with post-critical SARS-CoV2 disease
Source: Front Nutr. 2023 Nov 21;10:1218266. doi: 10.3389/fnut.2023.1218266 (PMC10702576; doi:10.3389/fnut.2023.1218266)
Supplement: Supplementary Table 1 — Baseline characteristics and morphofunctional assessment parameters, according to sex. [file Table_1.DOCX]

**SUPPLEMENTAL TABLES**

**Supplemental Table S1**. Baseline characteristics and morphofunctional assessment parameters, according to sex.

|  | **All** | **Male** | **Female** | ***p* value** |
| --- | --- | --- | --- | --- |
|  | ***N=75*** | ***N=58*** | ***N=17*** |  |
| **Demographic variables** |  |  |  |  |
| Age (years) | 62±12.0 | 62.2±12.9 | 57.9±9.4 | 0.249 |
| Diabetes Mellitus (%) | 21(28.0) | 17(29.3) | 4(23.5) | 0.641 |
| Arterial hypertension (%) | 38(50.7) | 28(48.3) | 10(58.8) | 0.444 |
| Dyslipidaemia (%) | 26(34.7) | 23(39.7) | 3(17.6) | 0.094 |
| Obesity by BMI ≥ 30 kg/m^2^ (%) | 34(45.3) | 25(43.1) | 9(52.9) | 0.356 |
| Obesity by FM ≥ 30% (men) or ≥ 40% (women) | 36(48.0) | 30(51.7) | 6(35.3) | 0.444 |
|  |  |  |  |  |
| **Nutritional Status** |  |  |  |  |
| Usual weight (kg) | 90.5±21.1 | 100.1±24.1 | 94.2±27.4 | 0.183 |
| Discharge weight (kg) | 99.1±24.7 | 90.5±4.3 | 90.4±3.9 | 0.728 |
| Weight loss (%) | 10.9±8.31 | 12.0±1.2 | 7.4±1.1 | 0.051 |
| BMI (kg/m^2^) | 31.1±6.4 | 30.2±8.4 | 34.2±9.1 | 0.137 |
| Malnutrition (GSA): | 75(100.0) |  |  | 0.695 |
| GSA-A (%) | 0(0.0) | 0(0.0) | 0(0.0) |  |
| GSA-B (%) | 41(54.7) | 31(53.4) | 10(58.8) |  |
| GSA-C (%) | 34(45.3) | 27(46.5) | 7(41.2) |  |
| Malnutrition (GLIM): |  |  |  | 0.523 |
| Normonutrition (%) | 13 (17.3) | 9(15.5) | 4(23.5) |  |
| Stage 1/ Moderate | 42(56.0) | 33(56.9) | 9(52.9) |  |
| Stage 2/Severe | 20(26.7) | 16(27.5) | 4(23.5) |  |
|  |  |  |  |  |
| **Complications and Need for Aggressive Therapies** |  |  |  |  |
| ICU stay (days) | 22.7±20.1 | 25.6±21.1 | 12.6±8.7 | 0.023* |
| Hospital stay (days) | 48.3±44.9 | 52.8±32.0 | 33.1±33.2 | 0.008* |
| Invasive Mechanical Ventilation (%) | 43(57.3) | 23(39.6) | 9(52.9) | 0.330 |
| Invasive Mechanical Ventilation (days) | 27.7±21.4 | 31.4±20.4 | 11.76±6.9 | 0.024* |
| Tracheostomy (%) | 27(62.8) | 25(43.1) | 2(11.7) | 0.014* |
| Manoeuvres prone (n) | 2.41±2.5 | 2.70±2.6 | 1.40±1.7 | 0.060 |
| Corticosteroid Therapy (%) | 74(98.7) | 57(98.2) | 17(89.45) | 0.586 |
| Home oxygen therapy after hospital discharge (%) | 39(52.0) | 25(43.1) | 8(47.0) | 0.773 |
| Rehabilitation after discharge (%) | 67(89.3) | 52(89.6) | 15(88.2) | 0.710 |
| FACIT scale | 40.1±13.1 | 41.6±12.1 | 35.0±15.1 | 0.102 |
| Barthel scale | 92.5±17.4 | 91.7±18.2 | 95.7±13.4 | 0.293 |
| **Bioelectrical Impedance variables** |  |  |  |  |
| PhA (º) | 4.9±1.12 | 5.02±1.2 | 4.86±0.9 | 0.609 |
| SPhA | -1.04(1.1) | -0.85 (0.99) | -1.41 (1.06) | 0.543 |
| TBW (L) | 45.6±9.7 | 47.5±9.4 | 39.3±8.4 | <0.001* |
| ECW (L) | 23.3±4.9 | 24.43±4.8 | 20.2±3.4 | <0.001* |
| TBW/FFM (%) | 76.1±3.9 | 76.2 (2.36) | 75.5 (1.82) | 0.713 |
| Na/K exchange | 1.15±0.2 | 1.16 (0.16) | 1.07 (0.14) | 0.042* |
| BCM (kg) | 28.9±8.6 | 30.0 (5.1) | 24.9 (3.82) | 0.028* |
| BCM/h (kg) | 16.9±4.6 | 17.3±4.6 | 15.3±4.2 | 0.102 |
| FFM (kg) | 59.9±11.7 | 62.2±11.1 | 51.9±10.3 | 0.001* |
| FFMI (%) | 20.5±2.9 | 19.1 (1.74) | 18.4 (1.70) | 0.217 |
| FM (kg) | 30.6±13.4 | 23.2 (7.37) | 29.0 (10.8) | 0.016* |
| ASMM (kg) | 23.6±5.9 | 8.85 (1.22) | 8.41(1.06) | 0.014* |
| ASMMI (kg/m^2^) | 8.07±1.56 | 8.14±1.4 | 7.78±1.9 | 0.308 |
| SMI (kg/m^2^) | 9.84±1.8 | 10.17±1.6 | 8.7±2.1 | 0.002* |
| SMM (kg) | 27.05±6.4 | 30.52±6.3 | 23.06±6.1 | <0.001* |
| **Nutritional Ultrasound**® **exploration** |  |  |  |  |
| RF-CSA (cm^2^) | 4.21±1.5 | 4.44±1.5 | 3.48±1.4 | 0.021* |
| RF/CSA/h (cm^2^/m) | 2.46±0.8 | 2.56±0.8 | 2.12±0.8 | 0.052 |
| RF/CSA/w (cm^2^/kg) | 4.65±1.35 | 4.84±1.3 | 4.03±1.4 | 0.060 |
| RF-CIR (cm) | 9.0±1.4 | 9.3±1.3 | 8.05±1.23 | 0.002* |
| RF-X axis (cm) | 3.68±0.6 | 3.82±0.5 | 3.27±0.75 | 0.002* |
| RF-Y axis (cm) | 1.32±0.4 | 1.34±0.4 | 1.25±0.5 | 0.498 |
| RF-SAT (cm) | 1.04±0.6 | 0.86±0.5 | 1.59±0.8 | <0.01* |
| T-SAT (cm^2^) | 1.89±0.9 | 1.74±0.8 | 2.32±1.0 | 0.033* |
| VAT (cm^2^) | 0.73±0.5 | 0.75±0.25 | 0.65±0.56 | 0.583 |
| VAT/h (cm/m) | 0.427±0.3 | 0.43±0.3 | 0.40±0.2 | 0.816 |
| VAT/kg (cm/kg) | 0.008±0.0 | 0.01±0.0 | 0.01±0.0 | 0.698 |
| **Functional tests** |  |  |  |  |
| R-HGS max (kg) | 24.7±12.3 | 26.9±12.1 | 18.1±10.0 | 0.019* |
| L-HGS max (kg) | 22.5±12.7 | 24.5±12.7 | 16.8±11.0 | 0.051 |
| TUG (s) | 8.49±2.7 | 8.56±3.1 | 8.23 ±1.1 | 0.841 |
| >12 (%) | 2(11.0) | 2(3.4) | 0(0.0) | 0.423 |
| <20 (%) | 0(0.0) | 0(0.0) | 0(0.0) | 1.0 |
| 6MW (m) | 365±107 | 366.1±108.6 | 361±106.0 | 0.985 |
| **Biochemical variables** |  |  |  |  |
| Vitamin D (mg/dL) | 22.3±9.2 | 23.1±9.1 | 21.2±9.6 | 0.506 |
| Albumin (g/dL) | 3.20±0.7 | 3.13±0.7 | 3.41±0.7 | 0.209 |
| Preabumin (mg/dL) | 27.3±12.5 | 27.1±12.0 | 27.9±13.1 | 0.900 |
| CPR (mg/L) | 26.9±50.1 | 28.9±51.0 | 13.1±13.6) | 0.335 |
|  |  |  |  |  |

Data are expressed as mean ± standard deviations or percentage. Groups were divided according to sex. Asterisk indicates significant difference between Male and Female groups, according to Student’s T-test (**p*<0.05). Chi squared test (or Fisher's exact test) was used for variables expressed as percentage (**p*<0.05). **Abbreviations**: PhA: phase angle; SPhA: Standardized phase angle; TBW: total body water; ECW: extracellular water; FFM: fat free mass; BCM: body cell mass; BCM/h: Standardized body cell mass; BMI: Body mass index; FM: fat mass; ASMM: appendicular skeletal muscle mass; SMI: skeletal mass index; ASMMI: appendicular skeletal muscle mass index. RF-CSA: rectus femoris cross-sectional area; RF-CSA/h: Cross-sectional area/height; RF-CIR: circumference of quadriceps rectus femoris; L-SAT: leg subcutaneous adipose tissue; T-SAT: total subcutaneous abdominal adipose tissue. VAT: total visceral adipose tissue. VAT/h: total visceral adipose tissue/height; VAT/w: total visceral adipose tissue/weight; CRP: C reactive protein; FM: Fat mass; HGS: Hand grip strength; SMI: Skeletal muscle index.

| **Supplemental Table S2**. Bioelectrical Impedance variables according to GLIM. | | | | | | | | | | | | | | | | | | | | | | | | | | | | | | | | | | |  |  |  |
| --- | --- | --- | --- | --- | --- | --- | --- | --- | --- | --- | --- | --- | --- | --- | --- | --- | --- | --- | --- | --- | --- | --- | --- | --- | --- | --- | --- | --- | --- | --- | --- | --- | --- | --- | --- | --- | --- |
|  | | | | | **Total** | | **Normonutrition** | | | | | | | | | | **Moderate malnutrition** | | | | | | | **Severe malnutrition** | | | | | ***p* value** | | | | | |  |  |  |
| PhA |  | | | 4.9±1.12 | | | |  | | | | 5.55±1.2 | | | | | |  | | | | | 4.9±0.9 | | | 4.8±1.1 | | | | |  | 0.020^*,a,b^ | | | | | |
| SPhA | | -1.00(1.1) | | | | | | | |  | | | -0.397±1.1 | | |  | | | | | | -1.44±1.0 | | | | | -1.2±1.0^c^ | | | |  | 0.002^*,a,b,c^ | | | | |  |
| TBW (L) |  | | | 45.6±9.7 | | | | |  | | | | | 49.3±8.7 | | |  | | | 43.2±10.3 | | | | | 45.9±9.6^c^ | | | | | |  | 0.012^*,a,b,c^ | | | |  |  |
| ECW (L) |  | | | 23.3±4.9 | | | | |  | | | | | 23.8±4.6 | | |  | | | 22.1±4.6 | | | | | 24.0±4.9 | | | | | |  | 0.380 | | | |  |  |
| FM (kg) |  | | | 30.6±13.4 | | | | |  | | | | | 38.2±20.0 | | |  | | | 27.5±9.7 | | | | | 29.7±12.0 | | | | | |  | 0.115 | | | |  |  |
| FFM (kg) |  | | | 59.9±11.7 | | | | |  | | | | | 64.6±10.2 | | |  | | | 57.5±11.8 | | | | | 59.9±11.9^c^ | | | | | |  | 0.001^*,a,b,c^ | | | |  |  |
| FFMI (kg/m^2^) |  | | | 20.5±2.9 | | | | |  | | | | | 22.3±2.2 | | |  | | | 19.4±2.7 | | | | | 20.3±3.1^c^ | | | | | |  | <.001^*,a,b,c^ | | | |  |  |
| BCM (kg) |  | | | 28.9±8.6 | | | | |  | | | | | 33.2±8.4 | | |  | | | 27.7±8.3 | | | | | 28.2±8.7^c^ | | | | | |  | 0.003^*,a,b,c^ | | | |  |  |
| BCM/h (kg/m) |  | | | 16.9±4.6 | | | | |  | | | | | 19.4±4.4 | | |  | | | 16.4±4.7 | | | | | 16.4±4.8^c^ | | | | | |  | 0.002^*,a,b,c^ | | | |  |  |
| Na/K exchange |  | | | 1.15±0.2 | | | | |  | | | | | 1.03±0.2 | | |  | | | 1.13±0.2 | | | | | 1.19±0.3 | | | | | |  | 0.093 | | | |  |  |
| TBW/FFM (%) |  | | | 76.1±3.9 | | | | |  | | | | | 76.2±3.9 | | |  | | | 74.9±3.4 | | | | | 76.6±4.3 | | | | | |  | 0.983 | | | |  |  |
| ASMM (kg) |  | | | 23.6±5.9 | | | | |  | | | | | 26.7±5.47 | | |  | | | 22.4±6.3 | | | | | 23.5±5.7^c^ | | | | | |  | 0.008^*,a,b,c^ | | | |  |  |
| SMM (kg) |  | | | 28.8±6.9 | | | | |  | | | | | 31.2±6.1 | | |  | | | 27.3±7.2 | | | | | 29.1±6.9^c^ | | | | | |  | 0.026^*,a,b,c^ | | | |  |  |
| SMI (kg/m^2^) |  | | | 9.84±1.8 | | | | |  | | | | | 10.8±1.7 | | |  | | | 9.24±1.8 | | | | | 9.87±1.8^c^ | | | | | |  | 0.008^*,a,b,c^ | | | |  |  |
| ASMMI (kg/m^2^) | | |  | | | 8.07±1.56 | | | | |  | | | | 9.19±1.4 | | | |  | | 7.56±1.5 | | | | | | | 7.95±1.5^c^ | |  | | | <.001^*,a,b,c^ |  |  |  |  |

Data are expressed as mean ± standard deviations or percentage. Groups were divided according to GLIM (normonutrition, moderate malnutrition, severe malnutrition). Asterisk indicates significant difference between groups, according to ANOVA (or Kruskal-Wallis U test) (**p*<0.05). ^a^Statistically significant differences between normonutrition and moderate malnutrition; ^b^Statistically significant differences between normonutrition and severe malnutrition; ^c^Statistically significant differences between moderate malnutrition and severe malnutrition. Chi squared test (or Fisher's exact test) was used for variables expressed as percentage (**p*<0.05). **Abbreviations**: PhA: phase angle; SPhA: Standardized phase angle; TBW: total body water; ECW: extracellular water; FFM: fat free mass; BCM: body cell mass; BCM/h: Standardized body cell mass; BMI: Body mass index; FM: fat mass; ASMM: appendicular skeletal muscle mass; SMI: skeletal mass index; ASMMI: appendicular skeletal muscle mass index.

**Supplemental Table S3.** Nutritional Ultrasound® variables according to GLIM.

|  | **Total** | | | | | | | | | **Normonutrition** | | | | | | | | | | **Moderate malnutrition** | | | | | | **Severe malnutrition** | | | | | | | | **P** | | | | | |  |  |  |  |
| --- | --- | --- | --- | --- | --- | --- | --- | --- | --- | --- | --- | --- | --- | --- | --- | --- | --- | --- | --- | --- | --- | --- | --- | --- | --- | --- | --- | --- | --- | --- | --- | --- | --- | --- | --- | --- | --- | --- | --- | --- | --- | --- | --- |
| **RF-CSA (cm2)** | 4.21±1.5 | | | | | | | | | 4.79±2.0 | | | | | | 3.82±1.2 | | | | | | 4.26±1.4 | | | | | | | | |  | 0.770 | | |  |  |  |  |  |  |  |  |  |
| **RF-CSA/h (cm2/m)** | 2.46±0.8 | | | | | | | |  | | | 2.79±1.1 | | | | | 2.23±0.6 | | | | | | 2.47±0.8 | | | | | | | 0.699 | | | | | | |  |  |  |  |  |  |  |
| **RF-CSA/w (cm2/Kg)** | 4.65±1.35 | | | | | | | | 4.74±1.9 | | | | | | | | 4.51±1.17 | | | | | | 4.74±1.26 | | | | | | | 0.142 | | | | | | |  |  |  |  |  |  |  |
| **RF-MC** | | 9.0±1.4 | | | |  | | | | | 9.20±1.7 | | | | | | | 8.79±1.3 | | | | | | 9.15±1.3 | | | | | |  | | | 0.905 | | | |  | | | | |  |  |
| **RF X-axis (cm)** | | 3.68±0.6 | | | | |  | | | 3.61±0.8 | | | | |  | | | | 3..64±0.5 | | | | | | 3.78±0.6 | | | |  | | | | 0.947 | | |  | | | | |  |  |  |
| **RF Y-axis (cm)** | | | | 1.32±0.4 | | | |  | | | | 1.57±0.5 | | | | | 1.22±0.3 | | | | | |  | | | | 1.27±0.4 | | | 0.181 | | | | | | | | |  | | | | |
| **RF-SAT (cm)** | | |  | | 1.04±0.6 | | |  | | | | | 1.31±0.9 | 0.99±0.5 | | | | | | |  | | | | | | | 0.94±0.6 | 0.488 | | | | | | | | |  | | | | |  |
| **A-SAT (cm)** | | |  | | 1.89±0.9 | | |  | | | | | 2.35±1.2 | 1.92±0.8 | | | | | | |  | | | | | | | 1.71±0.8 | 0.383 | | | | | | | | |  | | | | |  |
| **A-VPAT (cm)** | | |  | | 0.73±0.5 | | |  | | | | | 0.70±0.3 | 0.79±0.4 | | | | | | |  | | | | | | | 0.73±0.6 | 0.212 | | | | | | | | |  | | | | |  |

Data are expressed as mean ± standard deviations or percentage. Groups were divided according to GLIM (normonutrition, moderate malnutrition, severe malnutrition). Asterisk indicates significant difference between groups, according to ANOVA (or Kruskal-Wallis U test) (**p*<0.05). ^a^Statistically significant differences between normonutrition and moderate malnutrition; ^b^Statistically significant differences between normonutrition and severe malnutrition; ^c^Statistically significant differences between moderate malnutrition and severe malnutrition. Chi squared test (or Fisher's exact test) was used for variables expressed as percentage (**p*<0.05). **Abbreviations**: RF-CSA: rectus femoris cross-sectional area; RF-CSA/h: Cross-sectional area/height; RF-CIR: circumference of quadriceps rectus femoris; L-SAT: leg subcutaneous adipose tissue; T-SAT: total subcutaneous abdominal adipose tissue. VAT: total visceral adipose tissue. VAT/h: total visceral adipose tissue/height; VAT/w: total visceral adipose tissue/weight.

| **Supplemental Table S4**. Biochemical variables according to SGA | | | | | | | | | | | | | | | | | | | | | | | | | | |
| --- | --- | --- | --- | --- | --- | --- | --- | --- | --- | --- | --- | --- | --- | --- | --- | --- | --- | --- | --- | --- | --- | --- | --- | --- | --- | --- |
| **Total All** | | | | | **SGA-B** | | | | | | **SGA-C** | | | ***p* value** | | | | | | | | | |  |  |  |
| **Vitamin D (mg/dl)** | |  | 22.3±9.2 | | |  | 21.7±9.8 | | 23.1±8.9 | | |  | | | |  | 0.681 | | |  | | |  |  |  |  |
| **Albumin (g/dL)** | 3.20±0.7 | | |  | | | | 3.15±0.6 |  | 3.26±0.8 | | | |  |  | | | 0.561 | | |  | | | | |  |
| **Prealbumin (mg/dL)** | |  | 27.3±12.5 | | |  | 28.2±14.1 | | 25.4±7.9 | | | |  | | | 0.642 | | | | | |  | | |  |  |
| **CPR (mg/L)** | |  | 26.9±50.1 | | |  | 21.3±27.1 | | 33.9±38.3 | | | | | | | 0.818 | | |  | | |  |  |  |  |  |

Data are expressed as mean ± standard deviations. Groups were divided according to SGA. Asterisk indicates significant difference between SGA-A and SGA-B groups, according to Student’s T-test (or Mann-Whitney test) (**p*<0.05). **Abbreviations**: CRP: C-reactive protein. SGA: Subjective Global Assessment.

| **Supplemental Table S5**. Correlation Matrix of BIVA variables, Nutritional Ultrasound parameters and functional test in post-critical COVID-19 outpatients. |
| --- |

|  |  |  |  |  |  |  |  |  |  |  |  |  |  | |  | | | | |  |  |  |  |  |
| --- | --- | --- | --- | --- | --- | --- | --- | --- | --- | --- | --- | --- | --- | --- | --- | --- | --- | --- | --- | --- | --- | --- | --- | --- |
| PhA | | **BCM/h** | | | **SMI** | **RF-CSA** | | **RF-Y axis** | | **R_HGS** | | | | | **TUG** | | |  |  |  |  |  |  |  |
| BCM/h |  | Pearson's r |  | 0.878 |  | — |  |  |  |  |  |  |  | |  | | | | |  |  |  |  |  |
|  |  | p-value |  | < .001* |  | — |  |  |  |  |  |  |  | |  | | | | |  |  |  |  |  |
| SMI |  | Pearson's r |  | 0.392 |  | 0.730 |  | — |  |  |  |  |  | |  | | | | |  |  |  |  |  |
|  |  | p-value |  | < .001* |  | < .001* |  | — |  |  |  |  |  | |  | | | | |  |  |  |  |  |
| RF-CSA |  | Pearson's r |  | 0.561 |  | 0.711 |  | 0.599 |  | — |  |  |  | |  | | | | |  |  |  |  |  |
|  |  | p-value |  | < .001* |  | < .001* |  | < .001* |  | — |  |  |  | |  | | | | |  |  |  |  |  |
| RF-Y axis |  | Pearson's r |  | 0.537 |  | 0.701 |  | 0.616 |  | 0.855 |  | — |  |  | |  |  | |  | | | |  |  |
|  |  | p-value |  | < .001* |  | < .001* |  | < .001* |  | < .001* |  | — |  |  | |  |  | |  | | | |  |  |
| R-HGS |  | Pearson's r |  | 0.612 |  | 0.681 |  | 0.450 |  | 0.658 |  | 0.550 |  | — | |  |  | |  | | | |  |  |
|  |  | p-value |  | < .001* |  | < .001* |  | < .001* |  | < .001* |  | < .001* |  | — | |  |  | |  | | | |  |  |
| TUG |  | Pearson's r |  | -0.428 |  | -0.271 |  | 0.020 |  | -0.300 |  | -0.289 |  | -0.546 | |  | — | |  | | | |  |  |
|  |  | p-value |  | 0.076 |  | 0.277 |  | 0.937 |  | 0.227 |  | 0.260 |  | 0.029 | |  | — | |  | | | |  |  |
| 6MWT |  | Pearson's r |  | 0.149 |  | 0.199 |  | 0.115 |  | 0.247 |  | 0.143 |  | 0.279 | |  | -0.791 | |  | | | |  |  |
|  |  | p-value |  | 0.358 |  | 0.217 |  | 0.478 |  | 0.174 |  | 0.428 |  | 0.128 | |  | 0.006* | |  | | | |  |  |

| Data are expressed as Pearson correlation coefficient (r). Asterisk indicates significant difference between groups, according to Pearson’s r (**p*<0.05). **Abbreviations**: RF-CSA: rectus femoris cross-sectional area; PhA: phase angle; BCM/h: Standardized body cell mass; SMI: skeletal mass index; TUG: timed get-up-and-go; 6MWT: 6 minute walk test, R-HGS: Right Handgrip strength.  **Supplemental Table S6**. Correlation Matrix of Nutritional Ultrasound parameters, complications and need for aggressive therapies in post-critical COVID-19 outpatients. | | | | | | | | | | | | | | | | | | | | | | | | | | | | |  |  |  |  |
| --- | --- | --- | --- | --- | --- | --- | --- | --- | --- | --- | --- | --- | --- | --- | --- | --- | --- | --- | --- | --- | --- | --- | --- | --- | --- | --- | --- | --- | --- | --- | --- | --- |
|  | | | | |  | |  |  | | | |  |  |  |  |  |  |  |  |  | |  | |  |  |  |  |  |  |  |  | |
|  | | | | | |  | | | | | | **RF-CSA** | | **RF-CSA/kg** | | **Y-Axis** | | **ICU Stay** | | | **Hospital**  **Stay** | | **IMV days** | | **Prone Maneuvers** | | **Barthel scale** | |  |  |  |  |
| RF-CSA/kg |  | | Pearson's r | | | | |  | | | | 0.762 |  | — |  |  |  |  |  |  | |  | |  |  |  |  |  |  | | |  |
|  |  | | p-value | | | | |  | | | | < .001* |  | — |  |  |  |  |  |  | |  | |  |  |  |  |  |  | | |  |
|  |  | | |  |  |  |  |  |  |  |  |  |  |  |  |  |  |  |  |  |  |  |  |  |  |  |  |  |  |  |  |  |
| Y-Axis |  | | Pearson's r | | | | |  | | 0.857 | | |  | 0.509 |  | — |  |  |  |  | |  | |  |  |  |  |  |  | | |  |
|  |  | | p-value | | | | |  | | < .001* | | |  | < .001* |  | — |  |  |  |  | |  | |  |  |  |  |  |  | | |  |
| ICU Stay |  | | Pearson's r | | | | |  | | -0.292 | | |  | -0.317 |  | -0.320 |  | — |  |  | |  | |  |  |  |  |  |  | | |  |
|  |  | | p-value | | | | |  | | 0.020* | | |  | 0.011* |  | 0.012* |  | — |  |  | |  | |  |  |  |  |  |  | | |  |
| Hospital Stay |  | | Pearson's r | | | | |  | | -0.311 | | |  | -0.228 |  | -0.348 |  | 0.764 |  | — | |  | |  |  |  |  |  |  | | |  |
|  |  | | p-value | | | | |  | | 0.013* | | |  | 0.072 |  | 0.006* |  | < .001* |  | — | |  | |  |  |  |  |  |  | | |  |
| IMV days |  | Pearson's r | | | | | |  | | | 0.026* | |  | -0.108 |  | -0.092 |  | 0.952 |  | 0.639 | |  | | — |  |  |  |  |  | | |  |
|  |  | p-value | | | | | |  | | | 0.885 | |  | 0.543 |  | 0.598 |  | < .001* |  | < .001* | |  | | — |  |  |  |  |  | | |  |
| Prone Maneuvers |  | Pearson's r | | | | | |  | | | -0.187 | |  | -0.232 |  | -0.139 |  | 0.579 |  | 0.320 | |  | | 0.293 |  | — |  |  |  | | |  |
|  |  | p-value | | | | | |  | | | 0.146 | |  | 0.070 |  | 0.291 |  | < .001* |  | 0.006* | |  | | 0.060 |  | — |  |  |  | | |  |
| Barthel scale |  | Pearson's r | | | | | |  | | | 0.186 | |  | 0.086 |  | 0.231 |  | -0.339 |  | -0.363 | |  | | -0.400 |  | -0.191 |  | — |  | | |  |
|  |  | p-value | | | | | |  | 0.159 | | | |  | 0.517 |  | 0.084 |  | 0.004* |  | 0.002* | |  | | 0.009* |  | 0.117 |  | — |  | | |  |
| FACIT scale |  | Pearson's r | | | | | |  | | | 0.038 | |  | 0.128 |  | 0.079 |  | -0.076 |  | -0.097 | |  | | -0.070 |  | -0.038 |  | -0.005 |  | | |  |
|  |  | p-value | | | | | |  | | | 0.767 | |  | 0.320 |  | 0.550 |  | 0.518 |  | 0.411 | |  | | 0.654 |  | 0.747 |  | 0.970 |  | | |  |
|  | | | | | | | | | | | | | | | | | | | | | | | | | | | | |  |  |  |  |

Data are expressed as Pearson correlation coefficient (r). Asterisk indicates significant difference between groups, according to Pearson’s r (**p*<0.05). **Abbreviations**: RF-CSA: rectus femoris cross-sectional area; RF-CSA/h: Cross-sectional area/height; IMV: invasive mechanical ventilation; ICU: Intensive Care Unit.

| **Supplemental Table S7**. Correlation Matrix of BIVA variables, complications and need for aggressive therapies in post-critical COVID-19 outpatients. | | | | | | | | | | | | | | | | | | | | | | | | | | |
| --- | --- | --- | --- | --- | --- | --- | --- | --- | --- | --- | --- | --- | --- | --- | --- | --- | --- | --- | --- | --- | --- | --- | --- | --- | --- | --- |
|  |  |  |  |  |  |  |  |  |  |  |  |  | |  |  |  |  | |  | |  |  |  | |  |  |
|  | |  | | **PhA** | | **BCM/h** | | **SMI** | | **ICU Stay** | | | **Hospital Stay** | | **IMV days** | | | **Prone Maneuvers** | | **Barthel** | | | |  |  |  |
| BCM/h |  | Pearson's r |  | 0.878 |  | — |  |  |  |  |  |  | |  |  |  |  | |  | |  |  |  | |  |  |
|  |  | p-value |  | < .001* |  | — |  |  |  |  |  |  | |  |  |  |  | |  | |  |  |  | |  |  |
| SMI |  | Pearson's r |  | 0.392 |  | 0.730 |  | — |  |  |  |  | |  |  |  |  | |  | |  |  |  | |  |  |
|  |  | p-value |  | < .001* |  | < .001* |  | — |  |  |  |  | |  |  |  |  | |  | |  |  |  | |  |  |
| ICU Stay |  | Pearson's r |  | -0.501 |  | -0.402 |  | -0.068 |  | — |  |  | |  |  |  |  | |  | |  |  |  | |  |  |
|  |  | p-value |  | < .001* |  | < .001* |  | 0.564 |  | — |  |  | |  |  |  |  | |  | |  |  |  | |  |  |
| Hospital Stay |  | Pearson's r |  | -0.605 |  | -0.512 |  | -0.146 |  | 0.839 |  | — | |  |  |  |  | |  | |  |  |  | |  |  |
|  |  | p-value |  | < .001* |  | < .001* |  | 0.212 |  | < .001* |  | — | |  |  |  |  | |  | |  |  |  | |  |  |
| IMV days |  | Pearson's r |  | -0.438 |  | -0.140 |  | 0.353 |  | 0.952 |  | 0.639 | |  | — |  |  | |  | |  |  |  | |  |  |
|  |  | p-value |  | 0.003* |  | 0.370 |  | 0.020* |  | < .001* |  | < .001* | |  | — |  |  | |  | |  |  |  | |  |  |
| Prone Maneuvers |  | Pearson's r |  | -0.442 |  | -0.292 |  | -0.029 |  | 0.650 |  | 0.526 | |  | 0.307 |  | — | |  | |  |  |  | |  |  |
|  |  | p-value |  | < .001* |  | 0.012* |  | 0.809 |  | < .001* |  | < .001* | |  | 0.048* |  | — | |  | |  |  |  | |  |  |
| Barthel |  | Pearson's r |  | 0.331 |  | 0.290 |  | 0.135 |  | -0.339 |  | -0.363 | |  | -0.400 |  | -0.191 | |  | | — |  |  | |  |  |
|  |  | p-value |  | 0.005 |  | 0.015* |  | 0.264 |  | 0.004* |  | 0.002* | |  | 0.009* |  | 0.117 | |  | | — |  |  | |  |  |
| FACIT scale |  | Pearson's r |  | -0.008 |  | -0.025 |  | -0.039 |  | -0.076 |  | -0.097 | |  | -0.070 |  | -0.038 | |  | | -0.005 |  | — | |  |  |
|  |  | p-value |  | 0.946 |  | 0.830 |  | 0.744 |  | 0.518 |  | 0.411 | |  | 0.654 |  | 0.747 | |  | | 0.970 |  | — | |  |  |
|  | | | | | | | | | | | | | | | | | | | | | | | | | | |

Data are expressed as Pearson correlation coefficient (r). Asterisk indicates significant difference between groups, according to Pearson’s r (**p*<0.05). **Abbreviations**: RF-CSA: rectus femoris cross-sectional area; RF-CSA/h: Cross-sectional area/height; PhA: phase angle; BCM/h: Standardized body cell mass; SMI: skeletal mass index; IMV: invasive mechanical ventilation; ICU: Intensive Care Unit.
